# Probing the Dynamic Strength of Biomolecular Interactions with Single-Cell Centrifugation

**DOI:** 10.1021/acscentsci.5c00648

**Published:** 2025-08-26

**Authors:** Hans T. Bergal, Koji Kinoshita, Wesley P. Wong

**Affiliations:** 1 Program in Cellular and Molecular Medicine, 1862 Boston Children’s Hospital, Boston, Massachusetts 02215, United States; 2 Department of Biological Chemistry and Molecular Pharmacology, Blavatnik Institute at Harvard Medical School, Boston, Massachusetts 02115, United States; 3 Department of Pediatrics, Harvard Medical School, Boston, Massachusetts 02115, United States; 4 Wyss Institute for Biologically Inspired Engineering, Harvard University, Boston, Massachusetts 02115, United States

## Abstract

Molecular interactions between receptors and ligands govern critical biological processes, from immune surveillance and T-cell activation to tissue development. However, current techniques for studying binding avidity often sacrifice throughput or precision. We introduce a high-throughput method for quantifying molecular and cellular binding kinetics using a centrifuge force microscope (CFM)—a compact imaging system integrated into a benchtop centrifuge. The CFM performs real-time force measurements on thousands of single cells in parallel, probing receptor–ligand interactions under controlled mechanical stress. To extend these capabilities, we developed a next-generation CFM with dual-channel fluorescence imaging that enables tracking of individual cell unbinding events. To demonstrate its utility, we profiled the binding mechanics of Bispecific T-cell Engager (BiTE) molecules, immunotherapeutic proteins that facilitate T-cell targeting of cancer cells. In cell–protein assays, we quantified the avidity of T and B cells interacting with BiTE-modified surfaces, revealing receptor-specific correlations between ligand concentration and bond strength. In cell–cell assays, we characterized BiTE-mediated adhesion between Jurkat and Nalm6 cells, demonstrating a time-dependent increase in avidity. By integrating force spectroscopy with fluorescence imaging, the CFM provides a high-throughput approach for investigating the mechanochemical principles underlying receptor-mediated interactions, with broad implications for biophysical chemistry, molecular recognition, and therapeutic development.

## Graphical abstract

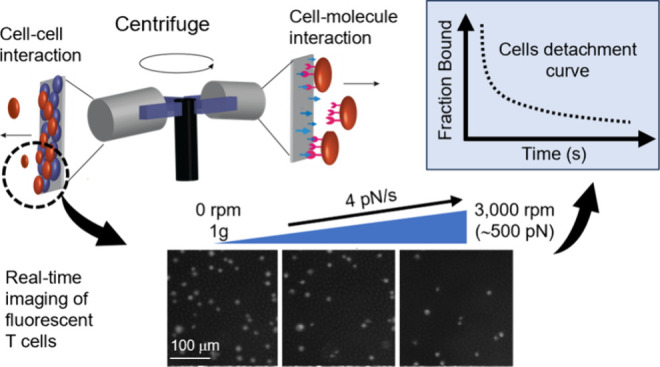

## 1 Introduction

Cell-cell binding is fundamental to many biological processes, from immune sensing to neuronal development. Beyond simple adhesion, receptor–ligand interactions can exert mechanical forces, leading to changes in cytoskeletal networks, opening of ion channels, and activation of signaling pathways.^1–4^ While many methods have been developed to measure the thermodynamic and kinetic parameters of individual protein–protein interactions,^5^ the interaction of single isolated proteins in solution may not directly translate to how they behave on the cell surface in coordination with other molecules.^6,7^ This is especially relevant under force, where bond rupture dynamics are highly dependent on the magnitude and duration of the applied mechanical load.^8^

A wide range of parameters, such as the identities of the proteins involved, the number of interactions, their spatial distribution, and time dynamics, can all contribute to the interaction strength between cells.^7,9^ For example, adhesion between an antigen-presenting cell (APC) and a T cell evolves from a weak interaction between a T-cell receptor (TCR) and major histocompatibility complex (MHC) proteins into a strong interaction composed of integrins organized into an immune synapse.^9^ T-cell responses can be further influenced by the affinity of TCRs,^10^ the number of receptors,^11^ and the applied force vector.^4^ Measuring cellular avidity in reaction to drugs, cell contacts, applied force, or over time would allow us to probe cellular behavior and gain critical insights into mechanisms of cell interaction and adaptation.

Cell–cell interactions often exhibit strong binding interactions, with lifetimes extending beyond feasible experimental timeframes.^12^ Moreover, the dynamic nature of the interactions that govern cell–cell adhesion requires precise, time-resolved measurements of off-rate kinetics to capture their behavior accurately.^13^ The mechanical force of cell adhesion has also been shown to play a critical role in immune processes like T-cell activation, proliferation, and cytotoxicity.^2^ Recent molecular force spectroscopy studies (also known as force spectrometry) have revealed the importance of force in T-cell receptor specificity^14^ and demonstrated long lifetimes under physiological loads.^11^ These findings highlight the need for quantitative methods that probe how molecular-scale mechanical forces regulate immune cell behavior.

Over the past decades, micromanipulation techniques—including atomic force microscopy (AFM),^15^ optical tweezers,^14^ and the biomembrane force probe (BFP)^16–18^ —as well as multiplexed systems such as hydrodynamic flow setups^19^ and acoustic force spectroscopy (AFS),^20,21^ have been successfully used to measure cell avidity.^22,23^ While micromanipulation methods offer high temporal and force resolution, they often measure cells one at a time, which slows data collection, limits the characterization of cell heterogeneity, and hinders their broader adoption.^24–26^ AFS and hydrodynamic flow systems enable parallel measurements but generally produce nonuniform force fields, require external calibration due to complex input-to-force mapping, and may exert unwanted torque on cells.

To overcome limitations in throughput, accuracy, and accessibility inherent to traditional single-cell force methods, we developed an approach based on a fluorescence-enabled centrifuge force microscope^27^ (CFM), capable of applying well-defined centrifugal forces to thousands of molecular interactions in parallel. The CFM, a microscope integrated into a benchtop centrifuge, combines real-time imaging with straightforward force quantification during centrifugation.^28^ Its use of high-resolution imaging and a uniform centrifugal force field enables consistent, parallel measurements across the entire field of view.^29,30^ In one of its earliest applications, molecules were tethered between a surface and beads, enabling the simultaneous characterization of hundreds to thousands of single-molecule interactions under force, thereby expanding the achievable throughput of single-molecule force spectroscopy.^27,28^

Here, we introduce a next-generation multichannel fluorescence CFM to quantify both cell-protein and cell–cell interactions under force (Figure 1A). Cell-protein interactions are quantified by attaching proteins of interest to a surface, allowing cells to bind, and then measuring bond lifetimes by monitoring the real-time response of each cell under applied centrifugal force. To extend this approach, we substitute the protein-functionalized surface with a cell monolayer, enabling the direct measurement of cell–cell binding strength. Measuring cell–cell avidity with the CFM allows us to study complex interactions under physiological receptor densities without the need for labor-intensive protein purification. By applying force we can quantify the strength of cell–cell adhesion, which reflects the cumulative strength of multiple receptor–ligand interactions—avidity rather than single-bond affinity.^31^ This enables us to track how adhesion evolves from initial weak binding events, such as TCR-MHC engagement, to the formation of a stable immune synapse. Our approach reveals dynamic insights that traditional methods like sequencing or proximity-based chemical tags often miss,^32^ and addresses the inherent difficulty of studying dynamic, force-dependent cell–cell interactions in their natural context.

**Figure 1:**
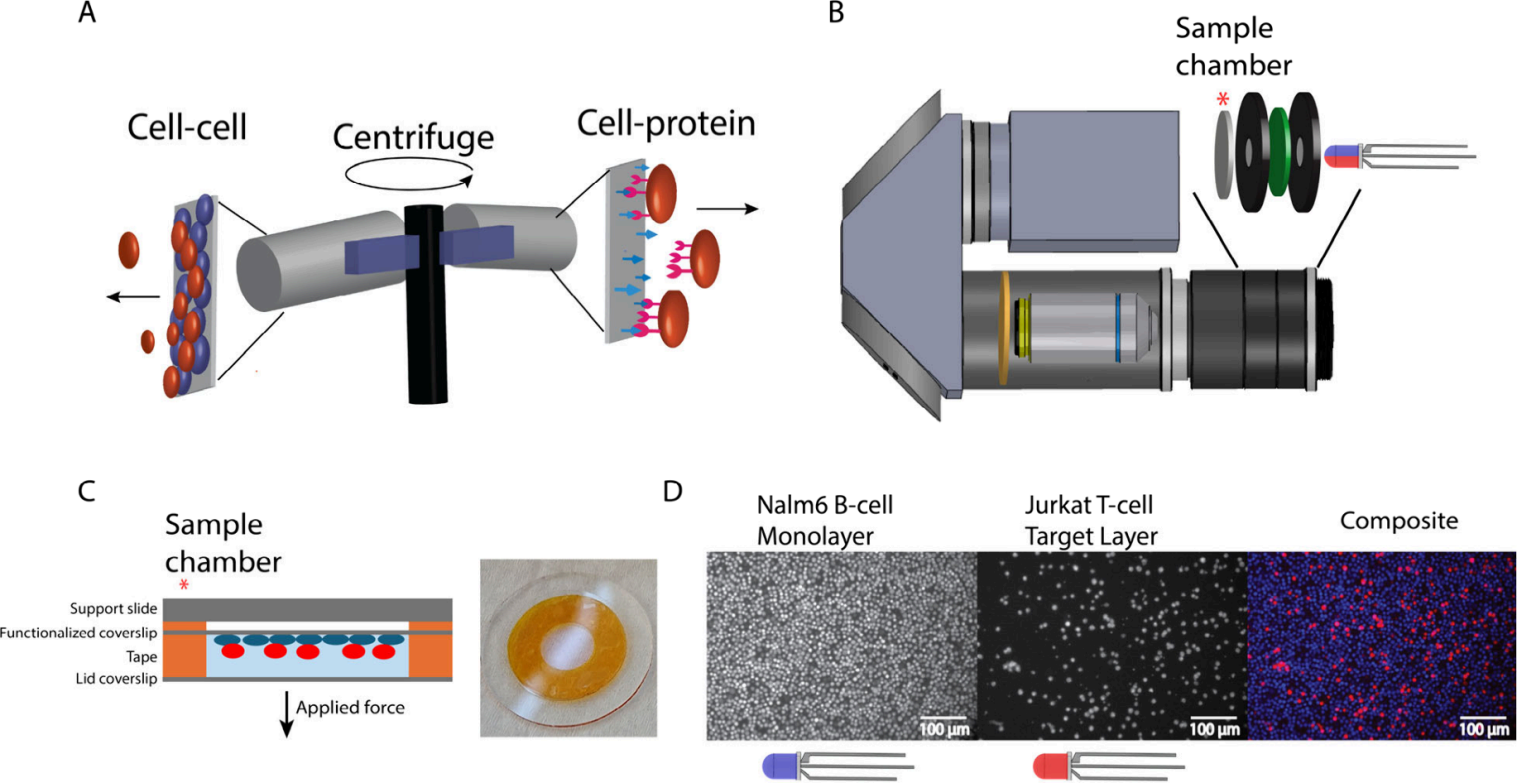
Overview of cell-avidity measurements using the fluorescence centrifuge force microscope (CFM). (A) Schematic of the measurement setup, where target cells (red) are placed onto a cell monolayer (blue) or a surface functionalized with target molecules. Centrifugal force is then applied, causing target cells to detach. (B) The fluorescence CFM setup advances previous designs by incorporating additional components, including an excitation filter (green), an emission filter (yellow), and a multicolor LED. (C) The sample chamber consists of two glass pieces held together with double-sided tape and an additional support slide. Cells are deposited onto a poly-l-lysine (PLL) functionalized coverslip before the sample chamber is sealed and loaded into the fluorescence CFM. (D) Fluorescent images showing labeled monolayer Nalm6 B cells and Jurkat T cells captured with the CFM (top view). Multicolor images are generated by cycling the LED color, ensuring each frame is illuminated by a single color. The images are then superimposed and false-colored to create a composite image.

To demonstrate biological applications, we investigated immune cell adhesion with an FDA-approved acute lymphoblastic leukemia drug,^33–35^ Blinatumomab, a bispecific T-cell engager (BiTE).^36,37^ Blinatumomab is a single-chain variable fragment of monoclonal antibodies containing a binding site recognizing CD3ε (L2K-O7 clone) and another site that recognizes CD19 (HD37 clone).^36^ The CD3ε domain of TCR complex is known to stimulate T-cell activation signals, initiating a cascade of intracellular events that are critical for T-cell proliferation, cytokine production, and cytotoxic activity, key processes in mounting an effective immune response.^38,39^ In contrast, CD19 is a well-established marker for B-cell leukemia and a common target in immunotherapy. Notably, the CD19-specific monoclonal antibody HD37 has been shown to inhibit B-cell activity and proliferation.^40^ The Blinatumomab molecules can simultaneously activate T cells via CD3ε and direct them toward CD19+ B cells.^41^ By looking at BiTE-mediated binding between Jurkat T cells and Nalm6 CD19+ B cells, we observed distinct time dynamics in the interaction profile that would be difficult to identify with other methods.

Our CFM-based approach provides the necessary statistics to quantitatively measure changes in cell adhesion across a wide range of conditions. Coupled with affordability and ease of use, our approach could broadly enable the study of critical cell processes, from fundamental cell biology to therapeutic cancer treatments like immunotherapy.

## 2 Methods

### 2.1 Materials

Nalm6 cells were purchased from ATCC (VA, USA). Jurkat E6-1 cells were a gift from Prof. Sizun Jiang. Poly-l-Lysine (PLL) coated coverslips (18 mm diameter, #1 thickness) were purchased from Neuvitro (H-18-PLL, WA, USA). Noncoated coverslips (18 mm diameter, #1 thickness) were purchased from VWR (48380-046, PA, USA). Blinatumomab recombinant antibody (MA5-41729) was purchased from Invitrogen (MA, USA). Soluble CD19 from Acro biosystem (CD9-H82F6, DE, USA) was used to block cell-bound CD19 from BiTE molecules. The OKT3 clone anti-CD3ε antibody was used to block CD3ε-BiTE interaction and shares 90% sequence homology with the anti-CD3ε domain of the BiTE molecule (Invitrogen 14-0037-82).^42^ Bovine Serum Albumin (BSA)-blocking solution was purchased from CANDOR Bioscience GmbH (BSA-block, Wangen, Germany). Jurkat and Nalm6 cells were grown in RPMI medium (ATC modification Thermofisher A1049101) supplemented with 10% Fetal Bovine Serum (FBS) (ATCC 30-2020) and 100 U/mL Penicillin-Streptomycin (Thermofisher 15070063). Amyl acetate was purchased from Millipore Sigma (W504009-500G, MA, USA). Nitrocellulose was purchased from Bio-Rad (#1620115). Phosphate Buffered Saline (PBS) buffer (10010-023, pH 7.4) was purchased from Thermo Fisher Science (MA, USA).

### 2.2 CFM Setup

The core setup of the centrifuge force microscope has been previously described,^29,43^ with updates for multicolor fluorescence provided in the updated parts list (Supplemental Table 1). A small microscope with a light-emitting diode (LED) light source, sample holder, objective, and camera is assembled (Figure 1B). The CFM weighs approximately 650 g and is less than 5 in. in length, making it compact enough to fit into a standard benchtop centrifuge bucket.^28^ The microscope is held in a custom 3D printed holder, which fits into the centrifuge (Heraeus X1R, Thermo Scientific). The centrifuge is modified with a fiber optic rotary joint and a computer control module, as specified in Yang et al., 2016.^28^ The camera signal is sent out from the centrifuge through a fiber optic cable with a rotary joint, allowing free rotation. Rechargeable batteries power the entire setup.

While single-channel fluorescent CFMs have been used to study colloids,^44^ we developed a novel CFM design with dual-channel fluorescence capabilities. A multicolor LED (red and blue) was positioned in transillumination beneath the sample chamber (Figure 1B), with a multi-bandpass excitation filter placed after the LED to filter out nonexcitation wavelengths. To optimize image quality, small apertures were added on either side of the excitation filter to block nonorthogonal light rays.

Upon LED illumination, distinct dyes labeling different cell types (Jurkat cells and Nalm6 cells) were excited before passing through the objective lens. A multi-bandpass emission filter, positioned behind the objective, selectively transmitted only the emitted fluorescence, allowing the Complementary Metal Oxide Semiconductor (CMOS) camera to detect cell positions (Supplemental Figure S1). The LED color was controlled by a microcontroller (Trinket M0, Adafruit) housed in the 3D-printed bucket holder, which was triggered by the CFM camera to alternate colors after each frame, enabling sequential dual-channel imaging (Figure 1D, Supplemental Figure S2).

To track cell dynamics, cell detachment curves were analyzed from 300 to 3000 rpm (13–1340 g), avoiding artifacts from centrifuge startup and nonlinear acceleration below 300 rpm. The computer control module in the centrifuge allowed specification of ramp protocols using stair-step commands, where the centrifuge accelerated to a defined speed before immediately increasing to the next step. In addition to internal centrifuge reporting, rpm was measured externally using a photodiode sensor (OPB732, TT Electronics, TX, USA), which detected the time between rotor revolutions based on a reflective marker attached to the rotor. The centrifuge was modified with a control module from Thermo Fisher Scientific to enable computer control. The force ramp protocol was implemented using WinMass (Thermo Fisher Scientific) centrifuge control software, which applied small incremental steps to achieve a linear force ramp (Supplemental Figure S3). The RPM controller script defined the loading rate based on the step size between successive commands. For quality control, the monolayer fluorescence channel was used to assess monolayer coverage and detect bubbles entering the field of view. Meanwhile, the target cell channel was analyzed using a custom image-processing pipeline, employing a Region-based Convolutional Neural Network (R-CNN) based on the YOLO5 algorithm^45,46^ to automatically identify and count cells in each frame.

### 2.3 Chamber Preparation

The CFM sample chamber is made with double-sided Kapton tape (Kapton PPTDE-3) sandwiched between one coated (nitrocellulose or PLL) coverslip and one blank coverslip as a lid (Figure 1C). Double-sided tape is cut as an annulus, with a 7.5 mm inner diameter and 18 mm outer diameter. To increase the volume and depth of the sample chamber, three layers of tape are stacked on the coverslip to create a chamber with a volume of ∼20 μL. After the sample is prepared with cells, the lid is attached. The sample chamber is mounted on a supporting slide glass with Kapton tape to give structural support during preparation and centrifugation (SI Howard Glass Co, B-270 Ø 25 mm, 0.9 mm thick).

### 2.4 Staining of Cells

Cells were grown to ∼1 million cells/mL in RPMI with 10% FBS and 50 U/mL Penicillin-Streptomycin at 37 °C with 5% CO_2_. Cells were counted using the Luna II automated cell counter (Logos Biosystems). For staining, 10 mL of cells in media were centrifuged at 400 g for 2 min. The media was removed, and the cells were resuspended in 1 mL of PBS. To stain, 1 μL of CellTrace CFSE dye (Invitrogen C34554) or 2.5 μL of CellTrace far red dye (Invitrogen C34564) (prepared to the manufacturer’s instructions in DMSO) was added. The cells were incubated at 37 °C for 20 min. The cells were spun down again, the buffer was removed, and the cells were resuspended in 10 mL RPMI+FBS. The cells were incubated for at least 20 min prior to imaging.

### 2.5 BiTE Molecule Attachment on the Nitrocellulose Coverslip

A layer of nitrocellulose was deposited on the surface by thinly spreading a solution of 1% (w/v) nitrocellulose in amyl acetate on the coverslip surface (18 mm diameter, #1 thickness). The cover glass was incubated in the oven (65 °C) for 10 min.

For BiTE adsorption, a 30 μL droplet of PBS (pH 7.4) containing a specified BiTE concentration (0 to 125 nM) was added to the nitrocellulose coverslip surface. The chamber was incubated for 60 min at room temperature to allow adsorption, and the entire chamber was gently soaked in 4 mL of PBS buffer once to remove the free BiTE molecules from the BiTE-attached sample surface. To minimize nonspecific binding on the coverslip’s surface, the chambers were filled with 20 μL of buffer A (10 mM HEPES, pH 7.4, with 150 mM NaCl and 20% (v/v) BSA blocking solution) for 30 min at room temperature.

Jurkat or Nalm6 cells were washed in buffer A and concentrated using centrifugation (400 g, 1 min). Cells were resuspended in buffer A to 10 million cells/ml (counted using the automated cell counter (Luna II, Logos Biosystem, South Korea)). For measurements, 5 μL of cells were added onto the BiTE-coated surface. The chamber was sealed with a lid coverslip on top.

### 2.6 Nalm6 Monolayer Attachment to the PLL Cover Glass

After staining, Nalm6 cells were concentrated to 40 million cells/ml in RPMI medium without FBS, and 30 μL were injected on the poly-l-lysine (PLL) coated coverslip surface. After allowing cells to adsorb onto the PLL-coated coverslip for 60 min, excess cells were removed by inverting the sample chamber and placing it upside down into a small well containing approximately 4 mL of PBS for 1 min. The chamber was then carefully lifted out—still upside down—and returned to its original orientation before adding fresh RPMI medium supplemented with FBS. The sample was incubated at 37 °C for at least 30 min prior to use. To preserve the sealing function of the Kapton tape, the top protective film was left in place until the lid cover glass was attached. A schematic of these procedures is provided in the Supporting Information (Supplemental Figure S4).

The chamber’s media was exchanged with 30 μL of buffer A mixed with the specified BiTE concentration. The cells were incubated (37 °C) for 5 min to allow BiTE to bind to CD19 receptors on the Nalm6 surface. After the incubation, 5 μL of buffer A + 10 million cells/ml Jurkat cells was added to the chamber. The chamber was sealed with the lid coverslip on top and incubated for the specified time before imaging. For blocking experiments, anti-CD3ε antibody (OKT3) was premixed with Jurkat cells for 5 min and OKT3 was also added to the monolayer at the same concentration before mixing. Jurkat cells for the cell–cell avidity experiment were prepared as described above.

To characterize the nitrocellulose surface, we applied a fluorescently labeled antibody (ThermoFisher A-11001) at varying concentrations in 30 μL droplets. After a 1 h incubation, we measured the residual fluorescence in each droplet on a plate reader (BioTek, Synergy H1) and compared it to a reference solution that had not contacted the coverslip. For this analysis, the decrease in fluorescence was attributed to the antibody adsorbing on the surface. Using the known stock concentration, fractional change in intensity, and droplet surface area, we calculated the average surface molecule density as a function of the applied concentration (Supplemental Figure S5). This measurement accounts for the total material putatively deposited, but does not take into account the orientation of the adsorbed molecules. Thus, it represents an upper limit in the number of available binding sites at each concentration. Due to this uncertainty, the sample preparation concentration is the parameter we used when labeling different conditions.

### 2.7 Imaging

The images collected from the camera were recorded with custom LabView software. Images were taken at 4 frames/second with an exposure time of 0.2 s per frame. Image size was 4096 × 2160, and a 20x objective (with the casing removed) was used (170 × 170 nm/pixel). Videos were analyzed using a custom processing pipeline that identified cells through a R-CNN based on the YOLO5 algorithm trained on a human-annotated data set.^45,46^ The total number of cells on each frame was counted. Small fluctuations in the number of cells detected from frame to frame could occur due to slight shifting of the field-of-view and slight differences in cell-object detection from frame to frame.

## 3 Results

### 3.1 Fluorescence Centrifuge Force Microscope: Instrumentation and Cell Assay Overview

To enable high-throughput single-cell measurements, we developed a centrifuge force microscope (CFM) with multichannel fluorescence capabilities based on a core design previously introduced by our lab.^28,43^ Briefly, the CFM is a small microscope in a 3D-printed holder that fits into a commercial centrifuge (Figure 1B).

An updated CFM was designed with dual-channel fluorescence imaging to monitor multiple cell types. A multicolor LED (red and blue) illuminated the sample, with multi-bandpass filters ensuring only fluorescence emissions reached the camera. A microcontroller synchronized LED color changes with image capture, allowing alternating frame detection of different cell types. Fluorescent labeling helped track cells, and a neural network-based image processing pipeline identified and counted them. The system also enabled quality control by monitoring the monolayer coverage and detecting bubbles.

After placing the cells onto the target surface, the chamber was sealed and loaded into the CFM (Figure 1C). A video recording was started, and simultaneously, the CFM was flipped as the microscope (Figure 2A, Supplemental Figure S6 and Supplemental Video File) was loaded into the centrifuge bucket. The flip changed the gravity from pushing the cells into the surface to pulling away from the surface, applying a low force of ∼0.3 pN per cell. We show four representative partial fields of view at key time points (Figure 2B): (1) the start, when the chamber is flipped and gravity pulls cells away (t = 0 s); (2) just before centrifugal force is applied, when adhesion frequency is measured (t = 120 s); (3) an intermediate point under centrifugal force (t = 145 s); and (4) the final point under maximum rotation speed (t = 250 s).

**Figure 2:**
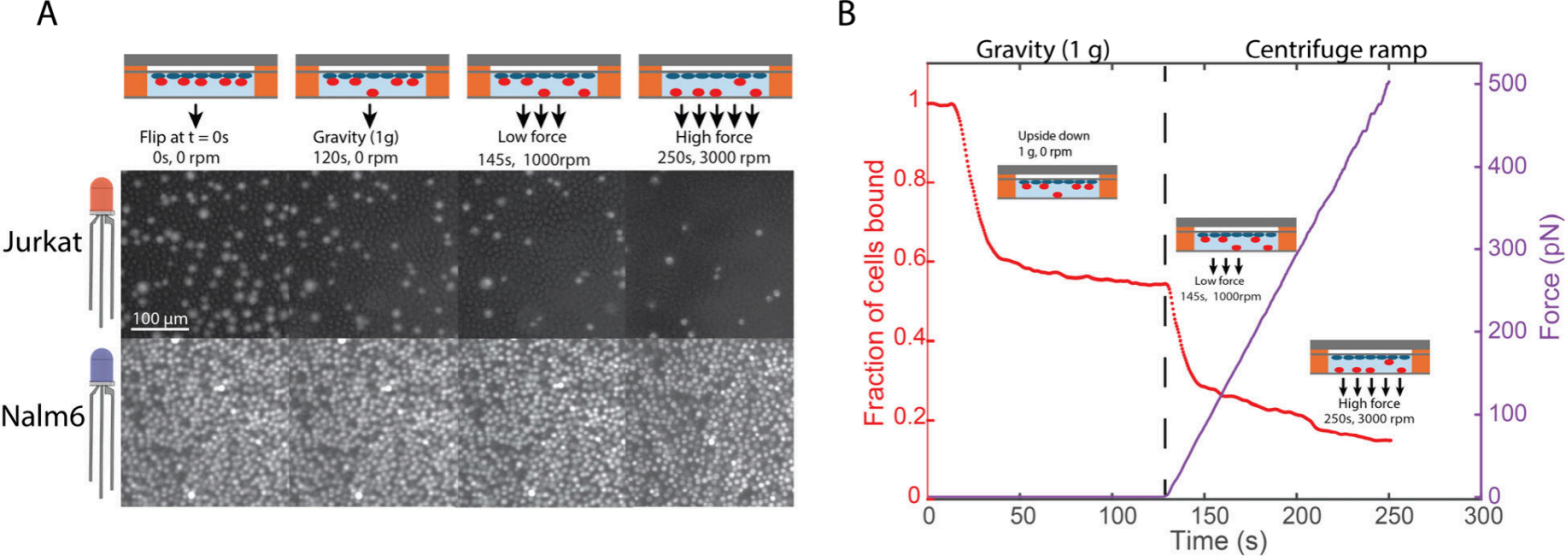
Fluorescence CFM detection of Jurkat T-cell detachment from a Nalm6 B-cell monolayer surface. (A) Partial field of view of Jurat T cells (top) and Nalm6 B-cells (bottom) from the CFM at different time points during centrifugation. At *t* = 0, the chamber was flipped upside down so gravity is pulling cells away, allowing nonadherent cells to leave the surface. At *t* = 120s, centrifugation began, and the force was ramped at a rate of 4 pN/s. As the force increased, Jurkat T cells unbound and disappeared from the field of view. Example video included in Supporting Information and Supplemental Figure S6. (B) Cell detachment curve (red, left axis) of Jurkat T-cell detachment showing the fraction of cells bound, initially under the force of gravity, then subsequently under a 4 pN/s force ramp. The corresponding applied force as a function of time (purple, right axis) was calculated from eq 1.

We generally observed an initial decrease in cell count during the interval under gravity, as nonadherent cells fell away due to gravity, before reaching a relatively steady level (Figure 2B, **gravity 1g section**). After 2 min, the centrifuge was activated and followed a linear force ramp protocol (Figure 2B, **centrifuge section**). An adhesion frequency was calculated as the ratio of cells remaining after the 2 min interval under gravity—prior to centrifuge activation—to the initial cell count. The adhesion frequency represents the fraction of cells exhibiting a minimum interaction strength, with the cells that detached due to gravity categorized as nonadherent.

The applied force on each cell is given by

1 $$F=m\times r\times \omega^{2}$$

Here, *F* is the applied force, *r* is the distance from the rotation axis, ω is the rotational speed, and *m* is the effective mass defined as *m* = *V*_cell_(*ρ*_cell_ – *ρ*_water_), where V is the volume of a single cell and ρ is the density. The volume and density of the Jurkat cell line we studied are well-documented in the literature and have previously been approximated as spheres with a diameter of around 10 μm and a density of 1.07 g/mL.^47–49^ Using these parameters, we calculated the applied force on each cell at each measured rotational speed (Figure 2B, **purple line**). By controlling the rotational speed, we obtained a linear force ramp between 2 to 16 pN/s, up to a maximum force of 500 pN per cell (Supplemental Figure S3).

### 3.2 Cell–Protein Measurements: Quantifying the Strength of BiTE-Immune Cell Interactions

Using the CFM method described above, we interrogated BiTE-immune cell interactions by attaching BiTE molecules to a surface, allowing cells to first bind before applying centrifugal force to measure their detachment lifetimes. By controlling protein identity and surface density, we initially validated the method to ensure reproducibility and characterize the specificity of binding interactions. We used the BiTE molecule Blinatumomab, which has one binding site for CD3ε and another for CD19, enabling measurements of both Jurkat T cells (CD3ε+ domain of TCR) and Nalm6 cells (CD19+) (Figure 3A).^36,50,51^ Each BiTE molecule can bind to only one receptor of each type at any given time.

**Figure 3:**
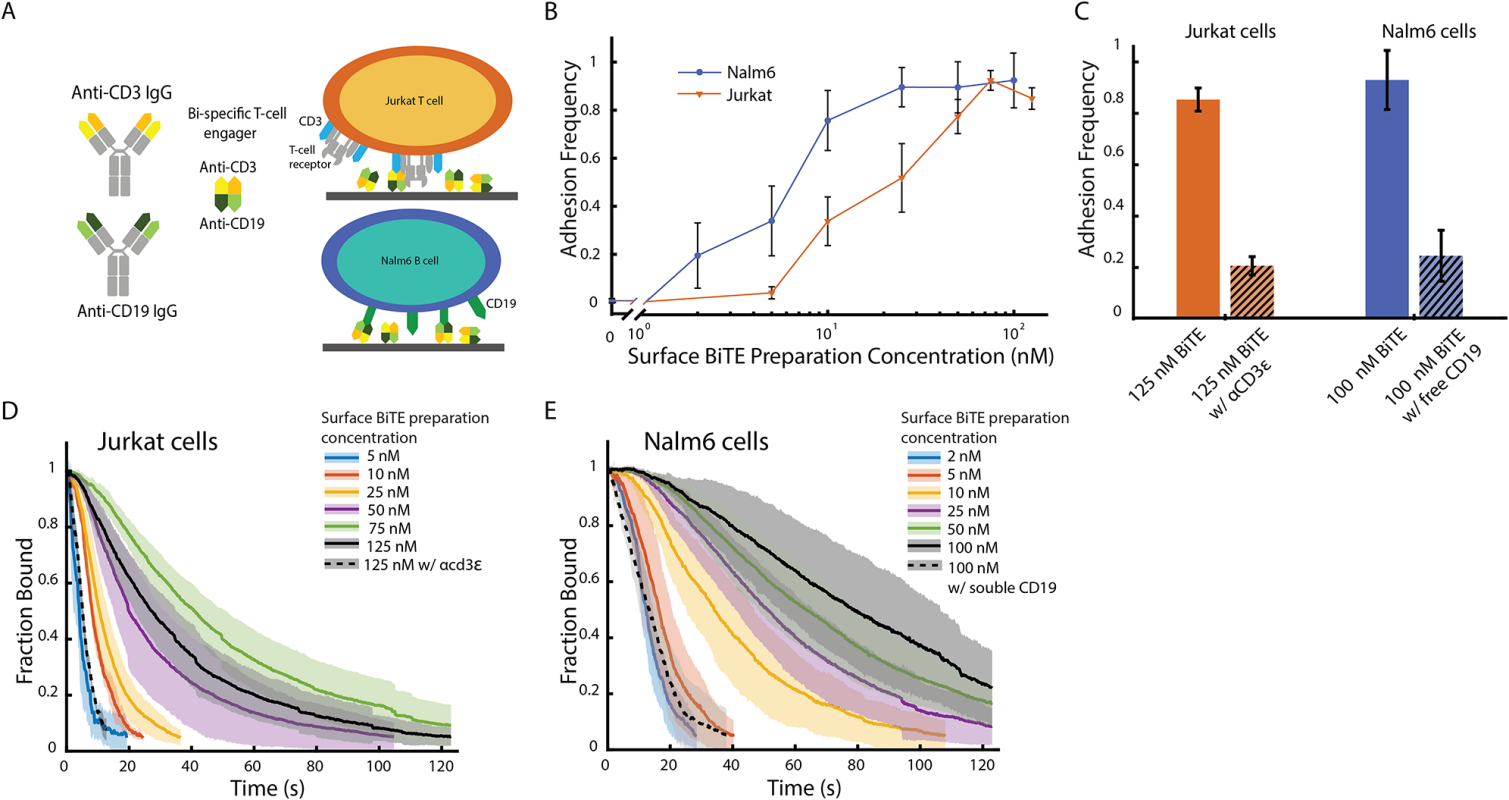
Jurkat T cells and Nalm6 B cell adhesion to BiTE functionalized surfaces. (A) Schematic image of cell attachment to the surface. BiTE (Blinatumomab) contains CD3ε and CD19 binding sites; it is randomly deposited on the surface and binds to JurkatT cells through the CD3 receptor and Nalm6 cells through CD19. (B) Jurkat (orange) and Nalm6 (blue) cell adhesion frequency vs BiTE preparation concentration on the glass surface after 2 min of gravity. Adhesion frequency is calculated as the number of cells after the 2 min gravity interval divided by the starting number of cells. Given concentration represents the concentration of BiTE solution the surface was prepared with. Number of trials in order of increasing concentration (Jurkat, Nalm6): *N*_trials_ = [5, 5, 3, 5, 5, 5, 5, 3], *N*_trials_ = [3, 4, 5, 3, 5, 5, 4, 3], Total cells observed: *N*_cells_ = [2582, 3070, 3805, 1818, 2827, 3389, 2555, 3186], *N*_cells_ = [3668, 3202, 1787, 5045, 3900, 4458, 4072, 2826], (C) The blocking of Jurkat cell and Nalm6 cell bindings on BiTE functionalized surfaces from addition of anti-CD3ε antibody OKT3 and CD19 proteins, respectively. Number of trials: *N*_trials_ = [5, 3, 4, 3]. (D) Cell detachment curves showing the fraction of Jurkat cells remaining bound on the surface with different BiTE concentrations in the presence of a 4 pN/s ramping force. Given concentration represents the concentration of BiTE solution with which the surface was prepared. For each concentration, at least three runs were averaged, with the standard deviation shown as the shaded region. Number of trials: *N*_trials_= [5, 3, 5, 5, 5, 5, 3], Total cells observed: *N*_cells_ = [128, 576, 2595, 3005, 4150, 3492, 584]. Fewer cells survive the 2 min 1 × *g* interval at lower concentrations, so fewer rupture events were observed. Full detachment curves are shown in Supplemental Figure S7. (E) Cell detachment curves showing the fraction of Nalm6 cells remaining bound on the surface with different BiTE concentrations under 4 pN/s ramping force. Number of trials: *N*_trials_ = [4, 5, 3, 5, 5, 4, 3], Total cells observed: *N*_cells_ = [622, 1263, 1406, 2518, 3023, 2369, 799]. Full detachment curves are shown in Supplemental Figure S8.

We saw a strong relationship between the concentration of BiTEs in the surface preparation and the resulting adhesion frequency (Figure 3B). When no BiTE was added, more than 99% of the cells fell off the surface during the gravity interval, indicating effective passivation with BSA blocking solution. For both cell types, increasing the BiTE concentration resulted in a higher adhesion frequency. At high concentrations, adhesion frequency plateaued, with more than 90% of the cells remaining after the initial gravity interval. Adding soluble OKT3 or soluble CD19 to the buffer competed with the BiTE-receptor interaction on their respective target cells (Figure 3C). This competition resulted in a significant decrease in cell adhesion for both Jurkat and Nalm6 cells (from ∼80–90% to ∼20%), supporting that the BiTE-receptor interaction drove the observed adhesion.

After the initial gravity interval of the CFM, the centrifuge was spun to apply a linear force ramp of approximately 4 pN/s at time *T* = 120 s (Figure 2B, purple line). Figure 3D–E illustrates the number of Jurkat or Nalm6 cells remaining as a function of time, normalized by the number of cells on the surface at the start of centrifuge acceleration. For each condition, multiple trials are averaged at each time point, with the standard deviation shown as a shaded band. Full detachment curves of each ramping experiment are available in Supplemental Figures S7 (Jurkat cells) and S8 (Nalm6 cells).

As the BiTE concentration increased, the lifetime of both cell types under force also increased, with cells requiring higher forces to be detached from the functionalized surface. Nalm6 cells exhibited longer lifetimes under force at a given BiTE concentration, possibly due to a greater number of receptors or to higher per-receptor affinity. For the Jurkat cells, cell detachment curves at preparation concentrations above 75 nM BiTE did not show increased binding strength, suggesting that the available binding sites were saturated. Saturation was observed in both the adhesion frequency and force ramping data, with a 125 nM BiTE concentration yielding a similar response to 75 nM BiTE. Blocking with OKT3 (aCD3ε) or soluble CD19 resulted in weaker adhesion, with the population rupturing at low forces (Figure 3D–E, dotted lines).

Each field of view contained approximately 500–1000 cells, and each trial took roughly 15 min. This high-throughput approach enabled us to capture over 25,000 single-cell unbinding events, allowing the construction of detailed cell detachment event distributions (Supplemental Figure S9). The method’s statistical power enables robust quantitative comparisons of binding behaviors across various conditions and cell types.

### 3.3 Kinetic Analysis of Cell Binding

To further investigate the dynamic strength of cell-BiTE interactions, we analyzed the kinetics of cell adhesion under increasing BiTE concentrations. At higher surface-BiTE concentrations, we observed both an increase in the number of cells that remained after the interval under gravity, and longer lifetimes under force of the cells that remained. Since both adhesion frequency and binding lifetime arise from molecular interactions such as the number of receptor–ligand bonds per cell, we analyzed their relationship to determine how they are governed by these shared molecular properties.

We found that the fraction of remaining cells, *f*(*t*), under a linear force ramp (Supplemental Figure S10), was well-described by a stretched exponential function of the form

2 $$f\left(t\right)=1-g+g\cdot \exp\left(-{\left(\frac{t}{\tau }\right)}^{\beta }\right)$$

A stretched exponential is a generalization of regular exponential decay that allows for more flexibility in modeling failure processes where the decay rate changes over time.^52^ In this case, the time constant τ provides a single metric that characterizes the population’s lifetime under specific ramping conditions, similar to how it would in a regular exponential decay. The β factor accounts for changes in the off-rate over time—here, the continuously increasing force accelerates the off-rate, effectively compressing the decay relative to a standard exponential. Finally, the offset term g represents the fraction of cells that remain bound to the surface.

Plotting the adhesion frequency against the fitted population lifetime τ for each cell detachment curve reveals a universal curve for a given force ramp and receptor type (Figure 4A-B). This relationship holds true regardless of surface concentration or variability between individual surface preparations, demonstrating a strong correlation between adhesion frequency and cell binding lifetime. Interestingly, blocking the binding site with a competitor reduced both the adhesion frequency and lifetime but preserved the relationship between them. The strong correlation suggests that a shared factor, likely the number of available receptors, governs binding. Intuitively, having more receptors increases the likelihood of cells adhering and enhances cell binding strength by providing more opportunities to form bonds.

**Figure 4:**
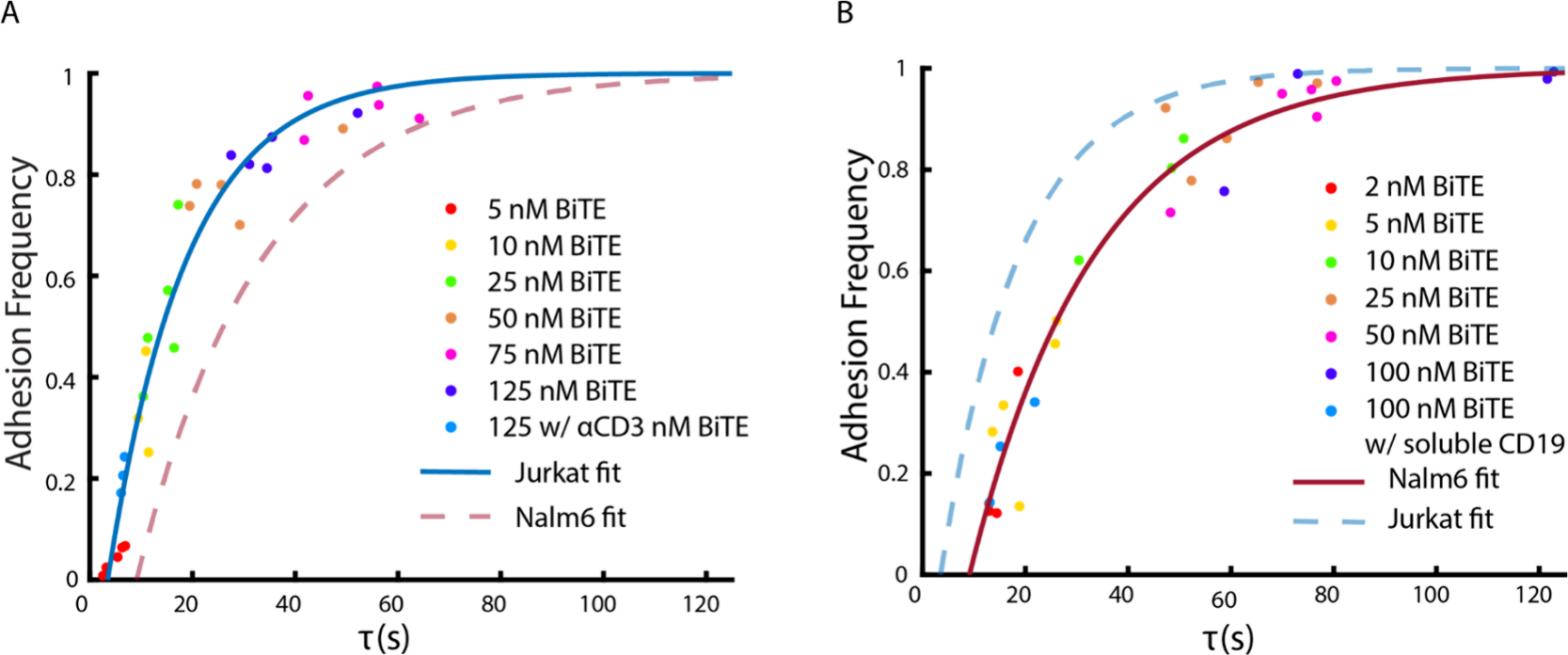
Binding analysis of surface functionalized with Blinatumomab BiTE to different cell types. (A) Each dot represents a single trial of Jurkat cells binding to BiTE, with surface concentration indicated by color. Adhesion frequency is calculated as the fraction of cells remaining bound after gravity relative to the initial number. The cell detachment curves under force (Figure 3D,E, ) are fitted to a stretched exponential function: $f\left(t\right)=1-g+g\cdot \exp\left(-{\left(\frac{t}{\tau }\right)}^{\beta }\right)$ The fitted lifetime parameter (τ) is plotted against adhesion frequency for each trial. To fit a universal curve to the data, a set of parametric equations based on the distribution of bonds per cell (given by λ) is used (see text). The predicted adhesion frequency is modeled as AF­(λ)=1–CDF. Poisson­(0, λ) and the predicted population lifetime is modeled by τ­(λ) = *kλ* + *x*_0_, with k and *x*_0_ as free parameters. The fitted curve (solid line) has parameters k = 15.4 *x*_0_ = 3.5 (RMSE: 0.07). The dotted line represents the analogous curve from the Nalm6 binding data in B for comparison. Number of trials: *N*_trials_ = 31, Total cells observed: *N*_cells_ = 23,232. (B) Nalm6 cells binding to a BiTE-functionalized surface, with data processed as explained in Figure 4A showing the relationship between fitted lifetime **τ** and adhesion frequency. The solid line shows the fit of the Nalm6 data (k = 24.5, *x*_0_ = 9.1, RMSE: 0.08), and the dotted line shows the Jurkat fit. Number of trials: *N*_trials_= 29, Total cells observed: *N*_cells_ = 28,958.

To model the relationship, we assume that the number of bonds formed on each cell follows a Poisson distribution characterized by the expected rate of bond occurrence, λ, and the number of bond occurrences, *x*,

3 $$P\left(x,\lambda \right)=\frac{\lambda^{x}\exp\left(-\lambda \right)}{x!}$$

The adhesion frequency represents the probability that at least one bond forms, ensuring survival during the gravity interval. This fraction is calculated using the complement of the cumulative distribution function (CDF) of the Poisson distribution evaluated at zero (*x* = 0), expressed as

4 $$\mathrm{AF}\left(\lambda \right)=1-{\mathrm{CDF}}_{\mathrm{Poisson}}\left(0,\lambda \right)$$

The fitted population lifetime (τ), measured during the force-ramp protocol, generally increases across trials with higher surface receptor densities, reflecting the greater number of bonds that can form under these conditions (Figure 4). For independent, noncooperative bonds loaded in parallel under a linearly ramping force, the lifetime of each cell scales approximately linearly with the number of bonds, provided the bond count is sufficiently large.^31,53^ If the number of bonds is Poisson distributed, the fitted population lifetime **τ** is approximately proportional to λ, scaled by a proportionality constant k. Additionally, an offset *x*_0_ is included to account for the lifetime not being zero when the adhesion frequency is zero. Thus, we can approximate the population lifetime by the expression τ­(λ) = *k* × λ + *x*_0_, where *k* and *x*_0_ are free parameters. The parameter k characterizes the strength of individual bonds, with a higher *k* value indicating a slower off-rate under these force-ramping conditions ().

Given the relationships for population lifetime (τ) and adhesion frequency (AF), we have established a set of parametric equations that relate adhesion frequency and population lifetime. By varying the surface concentration, we effectively sample different values of λ. These relationships were used to fit the experimental data, with *k* and *x*_0_ as the two free parameters representing aspects of individual receptor lifetimes and receptor densities. The fitted curves for Jurkat and Nalm6 cells were overlaid on the respective data sets (Figure 4A-B).

These universal curves differ between the two cell types, with the observed differences in adhesion strength potentially reflecting differences in the affinity of specific receptors. The fits suggest that the CD19-BiTE interaction is stronger than the CD3ε-BiTE interaction (CD19: *k* = 24.5, CD3ε: *k* = 15.4) as the Nalm6 cells (CD19+) survive longer at a given adhesion frequency than the Jurkat cells (CD3ε+). This trend is consistent with literature values for the dissociation constants, with a much stronger reported *K*_d_ for the interaction between Blinatumomab BiTE and Nalm6 than between BiTE and purified human T cells (1.49 × 10^–9^ M vs 2.6 × 10^–7^ M).^36^ It is important to note that our CFM cell adhesion measurements represent an aggregate signal influenced by multiple factors, including receptor affinity, receptor density, membrane contact area, receptor spatial distribution, and cell mechanical compliance. Thus, the observed differences in cell avidity may also reflect additional contributions from properties such as cell stiffness. Future studies could systematically investigate these additional parameters, potentially incorporating detailed analyses of cell shape and deformation at the single-cell level.

When measuring only one receptor type at a time, the model enables comparison of relative binding strengths between different receptors without requiring explicit measurement of receptor density on the cells.

We also demonstrated the ability to explore different force loading rates with the CFM (Supplemental Figure S12). Ramp protocols were created to apply various loading rates between 2 pN/s and 16 pN/s. The Jurkat T cell-BiTE interaction was measured over a range of loading rates at 10 nM BiTE, balancing statistics and low bond/cell number. The off-rate at a given force was calculated based on the number of cells that detached at a given force, normalized by the number of cells available and scaled by the time interval.^54,55^ As expected, the off-rate increased as the force increased. However, the observed off-rate plateaued as the number of cells decreased, possibly due to an increase in the fraction of multiply bonded cells. The technique demonstrates the CFM’s capability for measuring cell unbinding kinetics at different forces and loading rates.

### 3.4 Cell-cell Measurements: Jurkat Cell Adhesion on Nalm6-BiTE Monolayer Surface

Next, we used the CFM to interrogate BiTE-mediated cell adhesion of Jurkat T cells to Nalm6 B cells, measuring the interactions of immune cells at the single-cell level. Nalm6 B cells were attached to a glass coverslip to form a dense monolayer, then incubated with BiTE at the specified concentration; T cells were then added on top. The cell chamber was first inverted to measure adhesion frequency, defined as the fraction of cells remaining after a 2 min gravity interval. The force was then increased at a loading rate of 4 pN/s, as previously described. Based on the results in Sections 3.2–3.3, which provide evidence that both cell types bind to a specific site of the BiTE molecule (Figure 3B-E), we hypothesized that the BiTE would act as a bridge between the two cell types.

Surprisingly, varying the BiTE concentration had little observable impact on adhesion frequency within the tested range (Supplemental Figure S13A). Additionally, the cell–cell adhesion assays exhibited a relatively high background binding rate (∼30%) despite the use of BSA blocking solution, in contrast to the lower background observed in the cell-surface assays. This may have been due to the presence of various proteins, lipids, and sugars on the surface of the monolayer cells, interacting specifically or nonspecifically with the Jurkat cells. High background levels and trial variability may have obscured the relationship between BiTE concentration and adhesion frequency.

In order to determine the impact of BiTE concentration on adhesion strength, we examined the lifetime of each cell under a linear force ramp of approximately 4 pN/s. From the resulting cell detachment curves (Figure 5A), we observed that increases in BiTE concentration up to 75 nM enhanced the adhesion strength between the two cell types. As a negative control, we preincubated Jurkat T cells with an anti-CD3ε antibody to block the BiTE-CD3ε interaction, which reduced adhesion back to the 0 nM control level (Figure 5A, back dashed curve). This data supported the key role that BiTE bridging played in driving the increase in adhesion strength.

**Figure 5:**
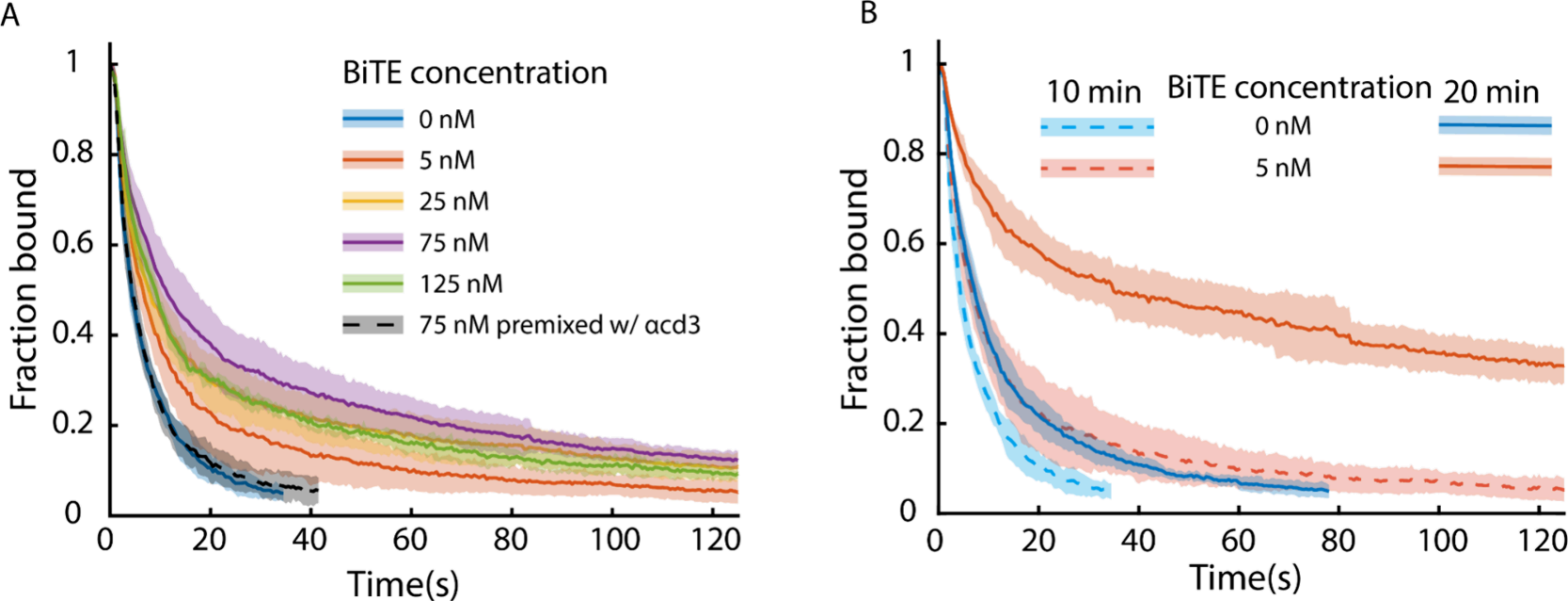
Jurkat T-cell strength on BiTE-treated Nalm6 cell monolayer. (A) Cell detachment curves of Jurkat T cells bound to a Nalm6 monolayer under 4 pN/s ramping force at different BiTE concentrations. Jurkat cells were incubated with a 10 min contact duration on the monolayer for all concentrations. Given concentration represents the concentration of BiTE in buffer mixed with the monolayer. For each concentration, at least three runs were averaged together, with the standard deviation presented as the shaded region. Number of trials: *N*_trials_ = [6,4,3,5,3,4], Total cells observed: *N*_cells_ = [1946,1578, 1447, 1909, 1036, 1138]. Full detachment curves under force in . (B) Cell detachment curves of Jurkat cells bound on Nalm6 monolayer at 10 and 20 min contact durations and at two different BiTE concentrations (0 nM, 5 nM) with 4 pN/s force ramp. Data listed: [0 nM 10 min, 5 nM 10 min, 0 nM 20 min, 5 nM 20 min]. Number of trials: *N*_trials_ = [6, 4, 4, 5], Total cells observed: *N*_cells_ = [1946, 578, 1946, 2650]. Full of cell detachment curves under force in Supplemental Figure S15.

Using the CFM’s parallel binding measurements, we investigated whether BiTE addition affects the time-dependent adhesion between Jurkat and Nalm6 cells. To do this, we varied the contact duration (i.e., the length of time that T cells are allowed to settle on the B cell monolayer prior to chamber flipping and the start of the measurement) and measured changes in binding strength. When the contact duration was extended from 10 to 20 min, it resulted in a marked increase in cell adhesion (Figure 5B). Under a linear force ramp, a much greater fraction of cells remained bound at high forces following 20 min of BiTEs incubation compared to 10 min. However, at the 20 min contact duration, adhesion showed little concentration dependence across the tested BiTE range (Supplemental Figure S16A). Notably, 5 nM BiTE at 20 min led to stronger adhesion than 75 nM BiTE at 10 min, underscoring the significant effect of contact duration. Despite the increased strength observed during ramping, increasing contact duration from 10 to 20 min caused minimal changes to the adhesion frequency (Supplemental Figure S13B).

To explore whether longer contact durations enhance adhesion strength through a cellular mechanism, we tested incubation with the activating anti-CD3ε antibody OKT3 (Supplemental Figure S16B).^56^ Since OKT3 binds exclusively to CD3 on Jurkat cells, it should not mediate direct adhesion to Nalm6 cells. As previously shown, OKT3 can block Jurkat-Nalm6 adhesion mediated by BiTE-receptor interactions after a 10 min contact duration (Figure 5A, black dashed curve). Interestingly, Jurkat cells incubated with only OKT3 for 20 min exhibited adhesion levels above the negative control, suggesting that some of the increased adhesion at 20 min in the BiTE experiments arises from T-cell activation rather than direct bridging alone. These results highlight the ability of the CFM to measure adhesion dynamics under different conditions to illuminate mechanistic differences underlying variations in T-cell binding.

## 4 Discussion

### 4.1 BiTE-Mediated T-Cell–B-Cell Avidity

Comparing cell adhesion mediated by BiTE molecules in the cell–cell versus the cell-surface assays reveal significant differences in their response to force as illustrated by their characteristic detachment curves (see Supplemental Figure S17). Specifically, we observed a lower overall adhesion frequency in the cell–cell experiments, but a higher proportion of cells maintaining adhesion at higher forces (Figure 3C–E, 5B, Supplemental Figure S13). In surface-functionalized experiments, cell binding was generally homogeneous, with higher adhesion frequencies correlating well with adhesion strength under force (Figure 4). In contrast, in cell–cell experiments, a significant fraction of cells detached immediately after flipping the chamber; however, a significant percentage of cells that remained after the gravity interval withstood the highest applied forces (Figure 5). The coexistence of a large population with weak adhesion and a smaller subset with much stronger adhesion suggests the presence of a distinct subpopulation with a different response to BiTE in the presence of Nalm6 B cells. Alternatively, this pattern may arise from substantial heterogeneity in surface receptor organization, potentially influenced by how cells land and establish contact. Further analysis using transcriptomic or proteomic profiling could help distinguish these possibilities.

Measuring cell–cell interactions between Jurkat and Nalm6 cells reveals minimal dependence of adhesion frequency (i.e., the fraction of cells remaining after the gravity interval) on BiTE concentration (Supplemental Figure S13). While surface functionalization can artificially increase receptor concentration, natural receptor abundance ultimately constrains cell–cell interactions. Consequently, beyond a certain threshold, increasing BiTE concentration may have little effect on adhesion, as the interaction is limited by the intrinsic densities of CD19 and CD3ε receptors. Given the relatively high background signal for the cell–cell binding assay, adhesion frequency alone cannot differentiate between different BiTE concentrations or contact durations. However, differences between conditions become apparent when force is applied. The strong dependence of cell binding on contact duration (i.e., the length of time between T cell addition and measurement) raises the question of what mechanism drives the increase. Jurkat cells binding to a BiTE-coated glass surface exhibited significantly lower retention under force than Nalm6-Jurkat at 20 min, with only 9 ± 7% remaining at 500 pN, even under saturated adhesion conditions (Figures 3D and 5B). This suggests that the increased avidity of Jurkat T cells on Nalm6 cells over time cannot be fully explained by BiTE-CD3 binding alone. Additionally, no dependence on contact duration was observed in the cell-surface assay (Supplemental Figure S18), suggesting that BiTE-CD3 binding reaches equilibrium within 10 min.

One possible mechanism for the increased avidity with longer contact durations is the lateral diffusion of CD3ε and/or CD19 within the plasma membranes of Jurkat T cells and Nalm6 cells, allowing for enhanced BiTE-mediated bridging. Previous findings show that TCR activation can induce the formation of TCR microclusters (TCR-MCs, 50–300 TCR molecules/cluster).^57^ These microclusters are known to be laterally mobile in the membrane, enabling the recruitment of additional receptors to the interface.^57,58^

Another potential mechanism involves CD3ζ-dependent activation of Jurkat T cells, which can enhance binding by recruiting additional protein binders or reorganizing existing interactions. Blinatumomab, a known T-cell activator, initiates signaling pathways that lead to the formation of an immunological synapse.^41^ The formation of this immune synapse is regulated not only by the lateral movement of TCR, LFA-1, and other membrane proteins but also by actin polymerization within the cytoskeletal network.^58,59^ Previous studies have shown that T-cell activation can induce actin-dependent centripetal movement of TCR and integrin microclusters, resulting in an immune synapse at the adhesion site.^12,58^

Supporting the activation mechanism, we observed a slight increase in avidity after a 20 min contact duration when Jurkat cells were incubated with the anti-CD3ε activating antibody OKT3 (Supplemental Figure S16B). Since OKT3 binds specifically to CD3+ T cells, any increase in binding is likely due to a cellular response rather than direct bridging. Notably, previous studies have reported a similar time-dependent increase in T-cell adhesiveness upon incubation with OKT3, consistent with our findings.^60,61^

Our findings using the Centrifuge Force Microscope indicate that the strong cell adhesion observed reflects a dynamic avidity response in Jurkat T cells interacting with Nalm6-BiTE cells. Notably, we observed significant changes in binding strength at low BiTE concentrations, aligning with clinically relevant concentrations used in patient treatments.^36,62^ Furthermore, the pronounced binding differences at longer contact durations became apparent only under force, underscoring the importance of measuring cell populations under force at specific time points to capture these critical dynamics.

### 4.2 Cell Avidity Force Measurement Tools

Built on a commercial microscope, the CFM enhances previous centrifuge-based techniques by providing improved temporal and force resolution for cell adhesion measurements. Centrifuge-based cell–cell adhesion assays have been used to quantify adherent cells before and after centrifugation, providing bulk measurements at a constant force.^63–69^ However, these approaches, without live imaging of cell detachments, require prior knowledge about the relevant force range, do not capture the full population of cell detachment curves, and only provide population-level data.

The fluorescence CFM overcomes these limitations by integrating real-time imaging to capture complete cell detachment curves and employing a dynamic force ramp that eliminates the need for prior knowledge of the relevant force range (Supplemental Table 2). The current CFM design operates at speeds up to 3,000 rpm (∼1,600 g), generating forces up to ∼500 pN for 10 μm cells while enabling parallel measurement of over 1,000 cells. A force ramp (1–16 pN/s) rapidly probes a broad force range to determine the relevant force thresholds for different interactions. The CFM can also perform constant force experiments by employing a fixed rotational speed, facilitating measurements at physiological force levels for over 30 min.

Real-time monitoring yields high-quality data with continuous quality control, such as tracking dynamic cell detachment from monolayer cells or functionalized surfaces (Figure 2A). The camera ensures proper monolayer adherence and detects disruptions, such as bubbles affecting cell-surface attachments. By enabling single-cell tracking, the CFM shifts experiments from bulk assays to single-cell analysis, capturing the entire distribution of cell lifetimes and identifying distinct cell populations. Incorporating multicolor imaging in the fluorescence CFM allows multiple cell types to be measured simultaneously or dyes to be included to monitor other processes, such as ion uptake.

Cell force spectroscopy assays provide a critical complement to existing methods for studying cellular interactions. Techniques like fluorescence imaging and chemical tagging provide detailed information about cell types, receptor identities, and receptor organization. However, standard fluorescence imaging approaches are typically limited to identifying binding versus nonbinding events rather than quantifying interaction strength.^32^ Similarly, while conventional flow cytometry is commonly used to measure equilibrium binding between proteins and cells, it generally lacks the temporal resolution to capture binding kinetics.^70^ In contrast, force-based cell adhesion assays are specifically designed to quantify binding strength between cells.

Our approach also complements other recently developed parallel cell avidity measurement techniques, such as AFS and hydrodynamic flow systems, which face several challenges including (1) nonuniform force fields, leading to spatial heterogeneity and unpredictable force vectors; (2) reliance on external calibration for force quantification and (3) high instrument and operational costs, which can limit accessibility.^20,25,26,71,72^ Centrifuge force microscopy, however, addresses these limitations and offers several advantages.

First, the centrifuge applies a uniform force field across the surface, with a straightforward relation between rpm and applied force (eq 1), virtually eliminating spatial differences in the direction and magnitude of force application across the sample. Force quantification is crucial in cell–cell measurements, where surface height and material properties could affect the force experienced by target cells under flow or acoustic waves. Unlike AFS, which relies on relative force calibration with standards like 10 μm polystyrene beads,^73^ centrifugation provides direct force quantification. Additionally, the CFM is a highly cost-effective and accessible solution, using standard lab equipment to enable the creation of a basic system for under $1,000–this reduces the cost barrier compared to most commercial optical or magnetic tweezers, AFM, or AFS systems, which can exceed $100,000.^28,74–76^ The CFM assay uses inexpensive, disposable coverslips, avoiding costly reusable chambers. This design supports rapid prototyping, diverse surface chemistries, and easier cleanup compared to AFS flow-through chambers. The protocol is straightforward, with centrifugation as the primary step, reducing the need for specialized expertise.

Further enhancements to the CFM could include increasing force ceilings and reducing variability from cell size and density differences. Faster centrifuges could achieve higher forces but would require imaging components that withstand high-g accelerations. Enhancing the density contrast between cells and medium could amplify forces but would necessitate careful osmotic regulation to prevent cell damage. Incorporating single-cell volume estimation could reduce force quantification error.^47–49^ Enhancing imaging parameters such as resolution, illumination strength, and field-of-view stability would further improve data quality and expand analytical capabilities.

### 4.3 Future Uses of CFM Cell Avidity Measurements

The CFM is well-suited for studying mechanical forces in cellular processes, including lymphocyte activation pathways.^77^ Parallel measurements complement optical trap studies by enabling broader experimental condition testing.^78^ Fluorescence imaging allows real-time monitoring of T-cell activation under physiological forces. Measuring adhesion strength under these conditions could provide insights into how initial weak forces can influence long-term avidity. Beyond force ramp experiments, the CFM can also perform long-duration force clamps (30+ minutes) to study lifetimes at physiological forces and catch bond behavior.^79^

Even with the complexity of cell–cell interactions, comparing rupture curves across different conditions could provide mechanistic insights into cell avidity. Assessing relative binding strength allows for differentiation between cell responses or types, such as identifying adhesion-related proteins in knockout libraries. Current CFM methods could reasonably screen ∼ 100 knockouts, correlating interaction strength with knocked-out proteins to dissect receptor contributions and uncover underlying mechanisms.

The ease and statistical power of CFM assays make them valuable for clinical applications. The CFM could assess immune cell binding in patient samples, correlating binding strength with disease state, age, genetics, transcriptomics, or drug response. Selecting cells based on specific binding strengths could aid immunotherapy development, particularly in cancer, where moderate receptor binding affinities are often optimal.^80^ The CFM’s precise force quantification enables targeted screening of desired binding strengths, making it a valuable tool for optimizing Chimeric Antigen Receptor (CAR) T cells. By measuring and selecting CAR T cells based on their mechanical binding profiles, the CFM could help improve efficacy and enhance therapeutic outcomes.

## 5 Conclusion

Our work introduces a high-throughput centrifuge force microscope (CFM) assay for quantifying the dynamic strength of receptor–ligand and cell–cell interactions at the single-cell level. By integrating multichannel fluorescence imaging, our advanced CFM enables real-time tracking of thousands of individual cell binding events in parallel, bridging the gap between single-molecule methods and high-throughput single-cell characterization. We demonstrate the utility of this approach through detailed characterization of immune-cell interactions mediated by therapeutic Bispecific T-cell Engager (BiTE) molecules, capturing critical time-dependent adhesion dynamics that traditional techniques often miss. Given its ease of use, accessibility, and throughput, the CFM provides a broadly applicable platform for investigating mechanochemical interactions across diverse fields, including chemical biology, biophysical chemistry, and therapeutic development.

## Supplementary Material







## Notes

Figures (S1–S18) for experimental setup and testing, additional controls for CFM binding experiments, raw detachment curves of CFM data reported in main text, CFM parts list, and CFM specifications table (PDF)
Nalm6 cells in a dense monolayer with Jurkat cells on top in a centrifuge with a real time RPM display in the top corner (MP4)
Transparent Peer Review report available (PDF)

## Abbreviations

CFM: centrifuge force microscope
BiTE: Bispecific T-cell engager
