# Supplementary material for: Probing the Dynamic Strength of Biomolecular Interactions with Single-Cell Centrifugation
Source: ACS Cent Sci. 2025 Aug 26;11(10):1946–58. doi: 10.1021/acscentsci.5c00648 (PMC12550632; doi:10.1021/acscentsci.5c00648)
Supplement: Supplementary file 1 [file oc5c00648_si_001.pdf]

Supplementary material: Probing the Dynamic Strength of Biomolecular Interactions with Single-Cell Centrifugation

Hans T. Bergal[1, 2†], Koji Kinoshita[1, 2†,] and Wesley P. Wong[1-4*]

[1]Program in Cellular and Molecular Medicine, Boston Children's Hospital; [2]Department of Biological Chemistry and Molecular Pharmacology, Blavatnik Institute at Harvard Medical School; [3]Department of Pediatrics, Harvard Medical School; [4]Wyss Institute for Biologically Inspired Engineering, Harvard University. † These authors contributed equally to this work. *Correspondence: Wesley P. Wong, Center for Life Sciences, 3rd floor, Boston Children's Hospital, 3 Blackfan Circle, Boston, MA 02215; e-mail: wesley.wong@childrens.harvard.edu

This PDF file includes:

Figs. S1 to S18
Tables S1 to S2

Includes background on experimental set up, raw data from centrifuge runs, and additional controls and conditions mentioned in the text. Tables include parts for instrumentation and limitations of method.

Other Supplementary Materials for this manuscript include the following:
Movies S1

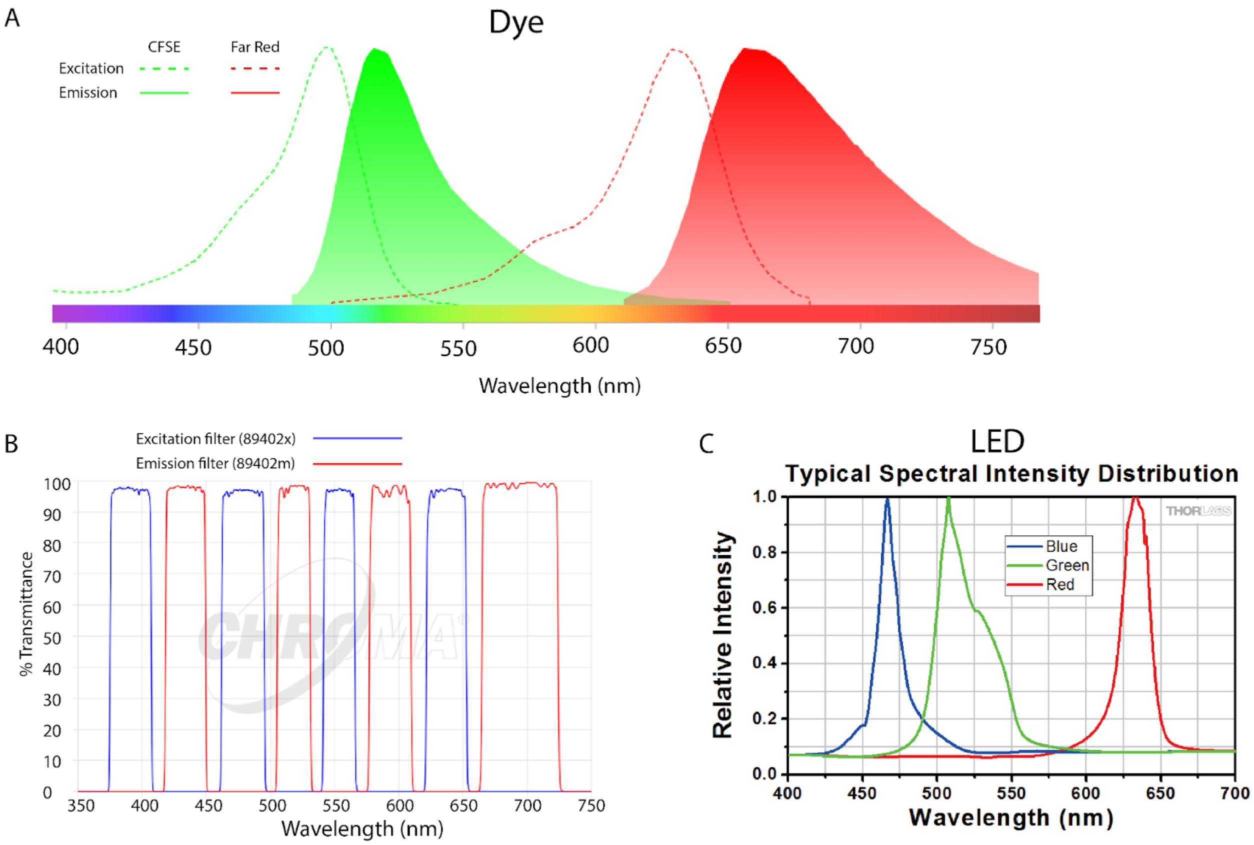

Figure S1. A. Excitation (dotted) and emission (filled) spectra for the two dyes used in the experiment (CellTrace CFSE, CellTrace Far Red). **B.** Percent transmittance spectra of excitation (89402x) and emission (89402m) filters. Data and filters from Chroma. **C.** LED emission spectra of three color RGB LED from Thorlabs. Data from Thorlabs. Full part list in Table S1.

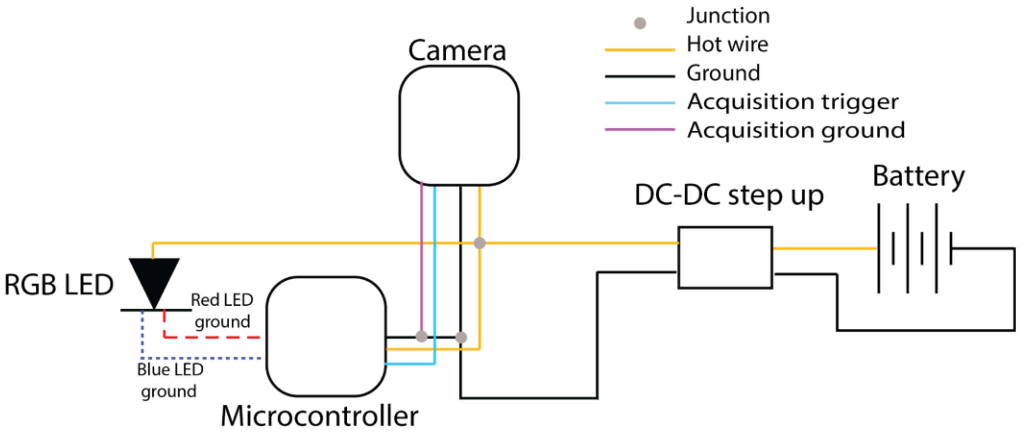

Figure S2 Circuit diagram for multicolor LED and microcontroller. Color is programmed by the microcontroller, which switches color based on the camera frame trigger. A detailed list of parts can be found in the **Supplemental table 1**. Similar to previous CFM designs, a 5V DC step-up converter is connected to the battery to maintain a constant voltage supply. This setup powers the camera, LED, and microcontroller. The camera's Pin 4 (opto-isolated output) is wired to an interrupt pin on the microcontroller, which detects a rising signal. The camera is configured to send a signal at the beginning of each frame. The LED's color is determined by grounding a specific colored lead, allowing current to flow and control the LED color. Each colored LED lead is connected to a pin on the microcontroller, which determines whether current flows and the resulting color. This configuration enables the LED color to sync with the camera's frame acquisition, illuminating only one color per frame. For protein-cell experiments, only one fluorescence channel was used.

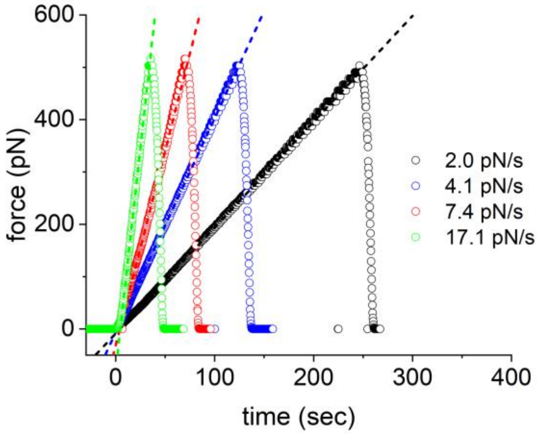

Figure S3. **Four different linear loading rates of CFM by controlling the operation program**. Loading rate calculation based on a spherical cell of diameter 10 microns, a buffer density of 1.00 g/ml, an estimated cell density of 1.07 g/ml, and a distance from the rotation axis of 15 cm.

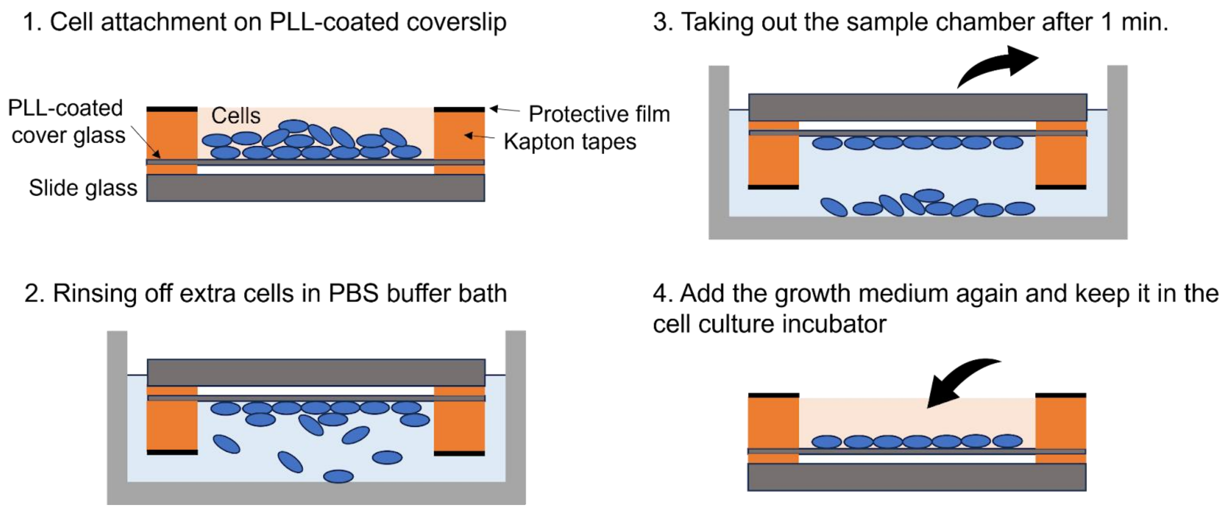

Figure S4. Schematic of Nalm6 cell monolayer formation on a PLL-coated coverslip.
The process involves four steps: **Step 1:** A suspension of Nalm6 cells in RPMI medium (without FBS) is added to the PLL-coated coverslip. **Step 2:** After a 60-minute incubation to allow cell adsorption, excess cells are removed by inverting the chamber and placing it upside down in a well containing ~4 mL PBS. **Step 3:** The chamber is then gently lifted out, remaining inverted to avoid disturbing the attached cells. **Step 4:** The chamber is returned to its upright position, and fresh RPMI medium with FBS is added. To preserve the seal formed by the Kapton tape, the protective film is left intact until the top cover glass is applied.

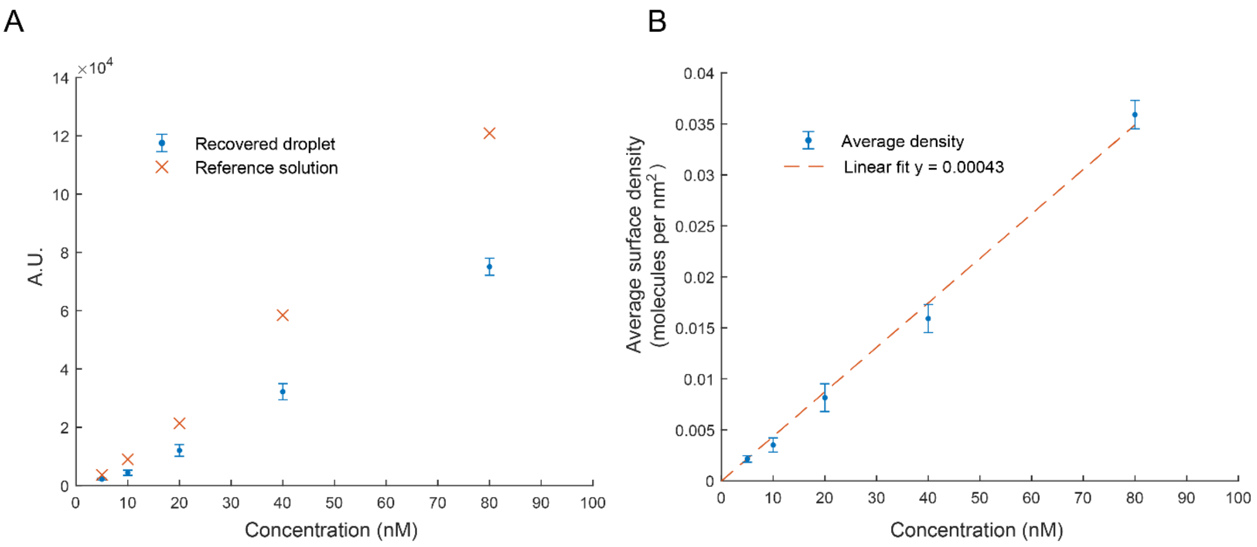

Figure S5. Surface density characterization A. Fluorescence intensity (Arbitrary Units A.U). measurements of a fluorescently labeled protein across a range of concentrations. Red '×' markers indicate reference solution measurements using a plate reader. Blue dots show measurements from recovered droplets that were first deposited onto a coverslip to allow protein adsorption, then recovered for measurement. Background fluorescence was subtracted using three PBS controls. Surface droplet

measurements were repeated three times and averaged. **B.** Using data from (A), the fraction of protein retained on the surface at each concentration was calculated based on the difference between recovered droplet and reference intensity measurements. From the known droplet volume and reference concentration, the number of surface-bound molecules was calculated. Assuming a droplet contact radius of 2.5 mm, the contact area was calculated and used to determine the average surface density (molecules/nm^2) as a function of input concentration. A linear fit ($y = 0.00043x$, $R^2 = 0.99$) describes the relationship within the tested range. This estimated density serves as an upper bound on the number of available binding sites, as it does not account for molecular orientation or packing. The relationship is expected to plateau at higher concentrations due to surface saturation, but the concentrations tested remain within the linear regime.

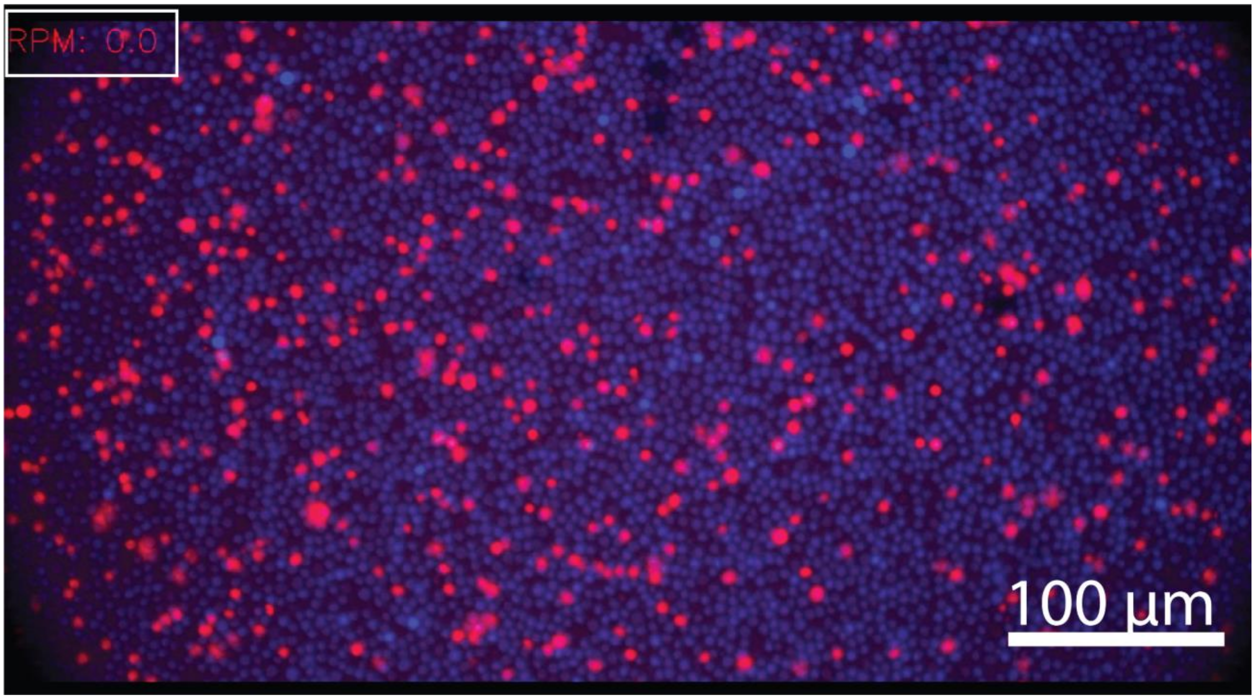

Figure S6. **Composite image of Nalm6 cells in a dense monolayer (blue) with Jurkat cells on top (red).** Full video of example included as a supplemental file. RPM in the top right corner shows the real time RPM of the centrifuge at time the image was taken. The video file is sped up 13x and images compressed to reduce file size.

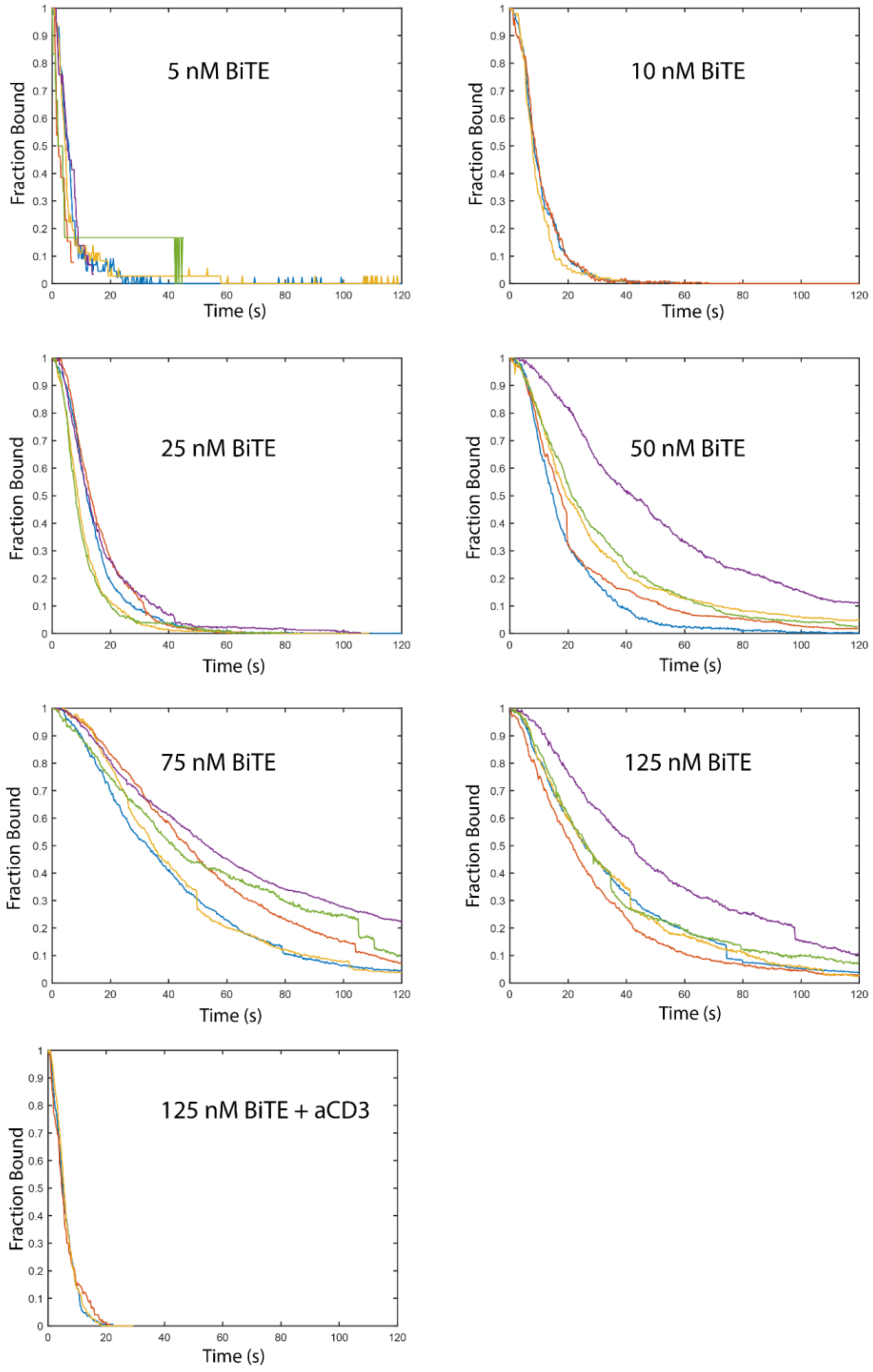

Figure S7. Individual trajectories for Jurkat cells on a BiTEs-coated surface with the indicated BiTE surface preparation. Total cells N_{cells} = [128,576,2595,3005,4150,3492,584]

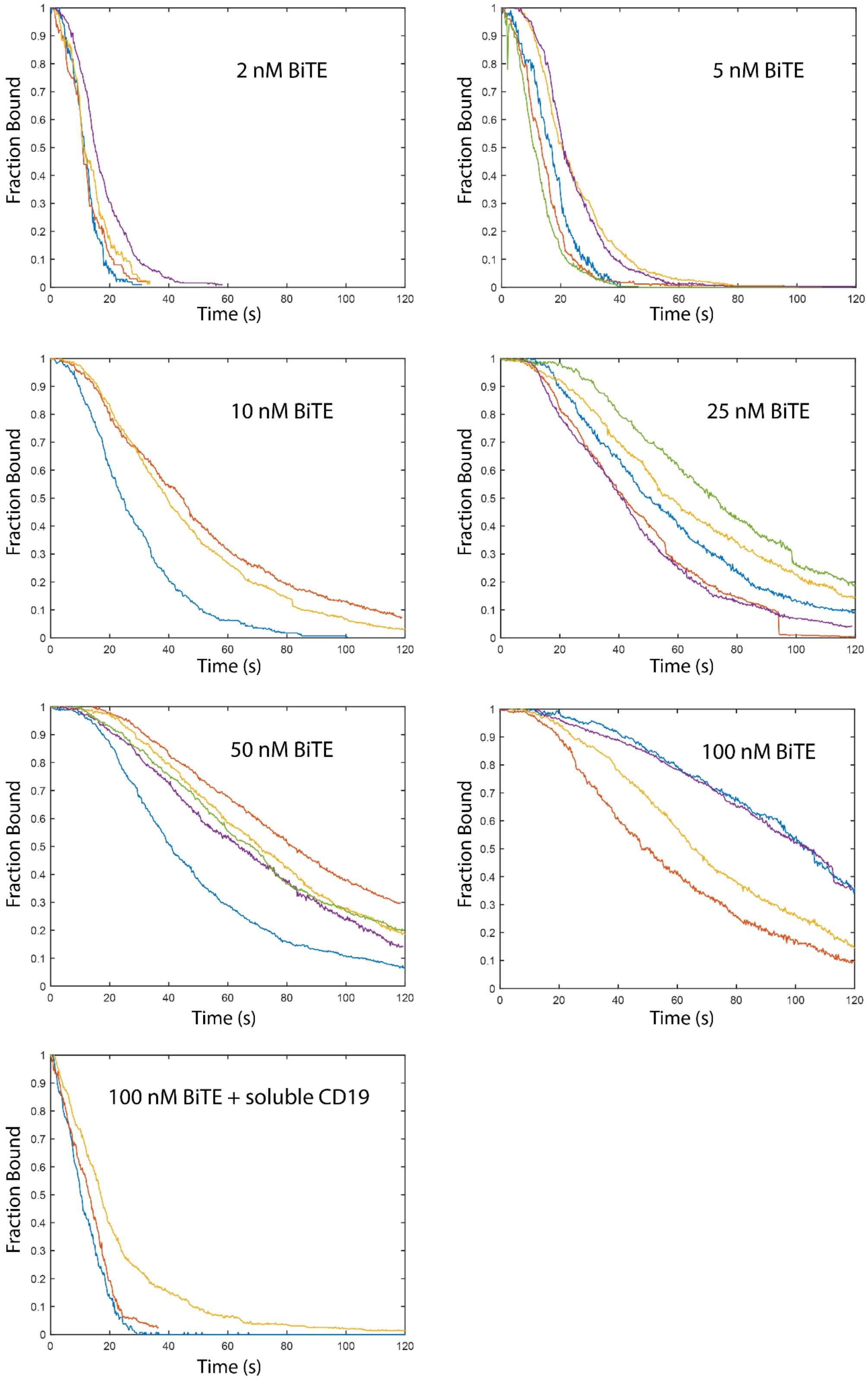

Figure S8. Individual trajectories for Nalm6 cells on a BiTEs-coated surface with the indicated BiTE surface preparation. Total cells N_{cells} = [622, 1263, 1406, 2518, 3023, 2369,799].

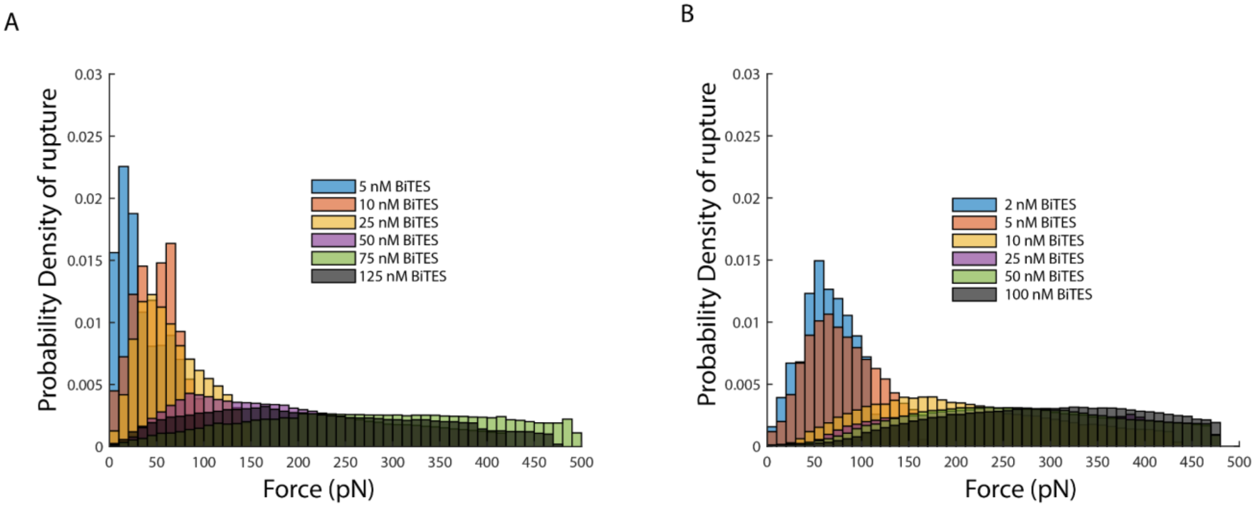

Figure S9. A. Rupture histogram for Jurkat cells binding to a BiTE functionalized surface prepared with a solution of the specified concentration. Total cells N_{cells} = [128,576,2595,3005,4150,3492]. Fewer cells survive the 2-minute 1xg flip at lower concentrations, so fewer rupture events were observed. **B.** Rupture histogram for Nalm6 cells binding to a BiTE functionalized surface prepared with a solution of the specified concentration. Total cells N_{cells} = [622, 1263, 1406, 2518, 3023, 2369]. The histograms are binned based on a nominal force calculation assuming constant cell density and size.

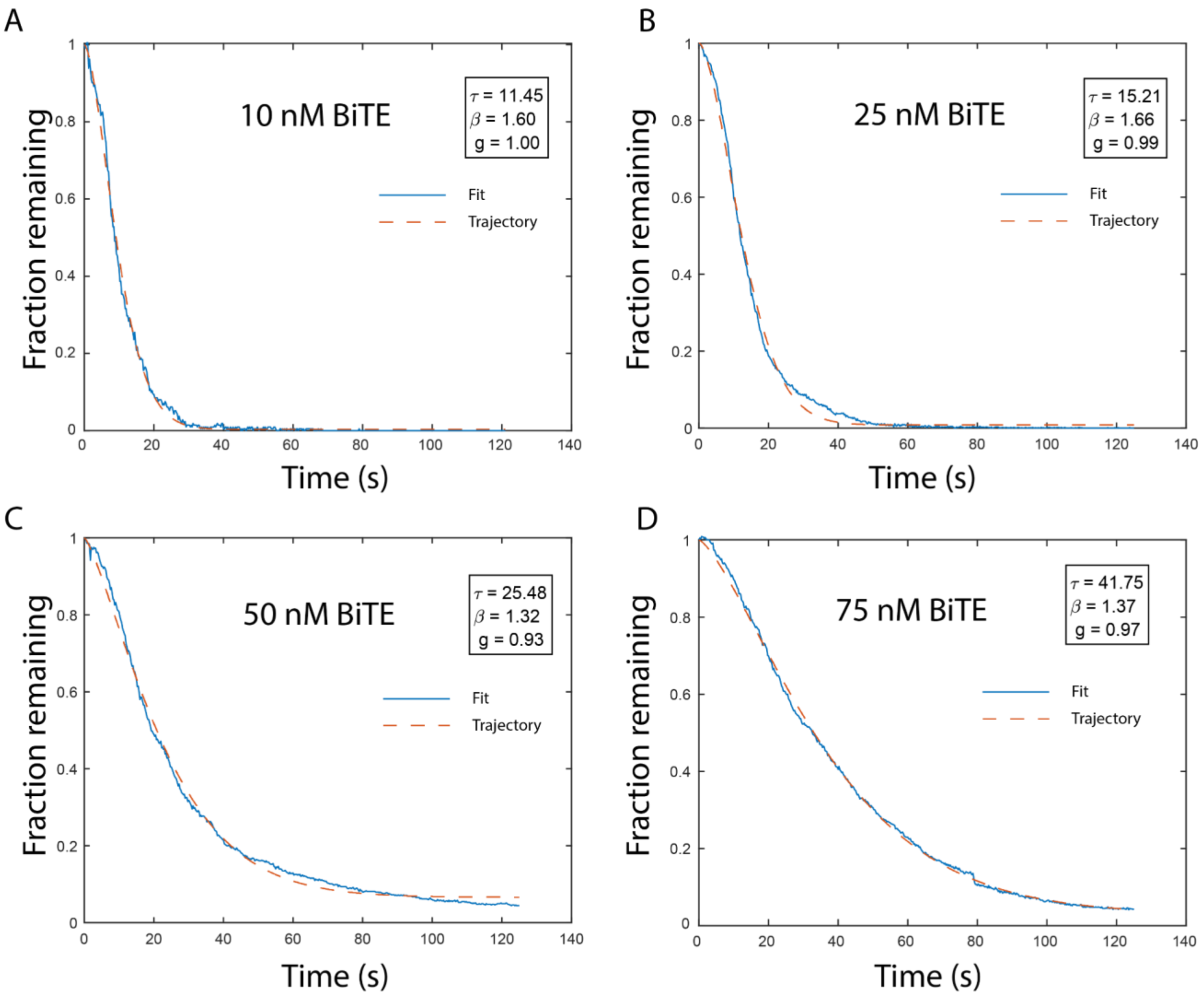

Figure S10. **Example stretched exponential fits 1-g+g*exp(-(t/τ)^β).** Trajectories are examples of Jurkat T-cells binding to BiTEs-functionalized surface prepared with the preparation concentration as indicated.

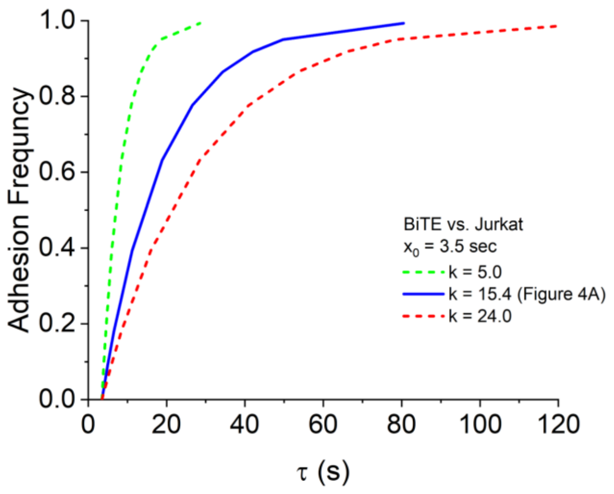

Figure S11. **Examples of different parameter k values for the adhesion frequency and population lifetime relationship in Figure 4A (main text).** The parametric equations (AF(λ) = 1 - CDF.$_{Poisson}$ (0,λ)) and population lifetime (**τ**(λ) = k * λ + x_0) were evaluated for different k values with x_0 set to 3.5. The increase of k value from 5.0 (green) to 24.0 (red) indicates a shallower slope, i.e., a slower off rate under our force ramping conditions.

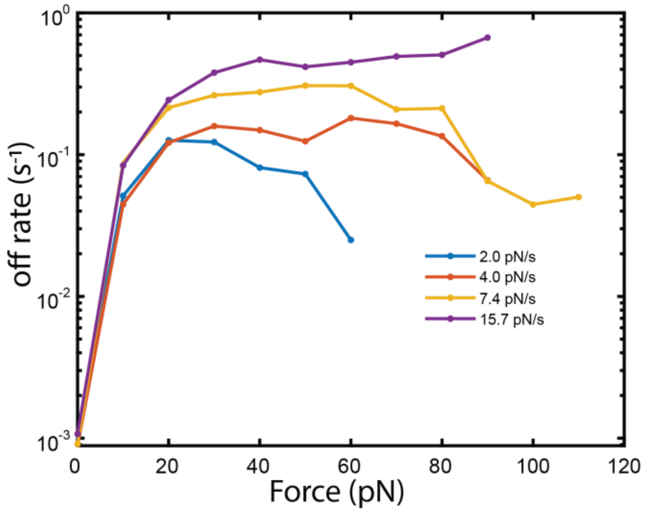

Figure S12. **Jurkat cells binding to a surface prepared with 10 nM BiTE solution were measured with different loading rates (1-16 pN/s).** The off rate at a given force was determined by normalizing the number of cells detaching at that force to the total number of available cells and scaling by the time interval [Evans 2009] [Dudko 2008]. The zero force off rate was calculated based on an interval during the flipping. Data is listed in terms of increasing loading rate. N_{trials} = [3,3,4,3], Total cells N_{cells} = [633,606,1147,557]

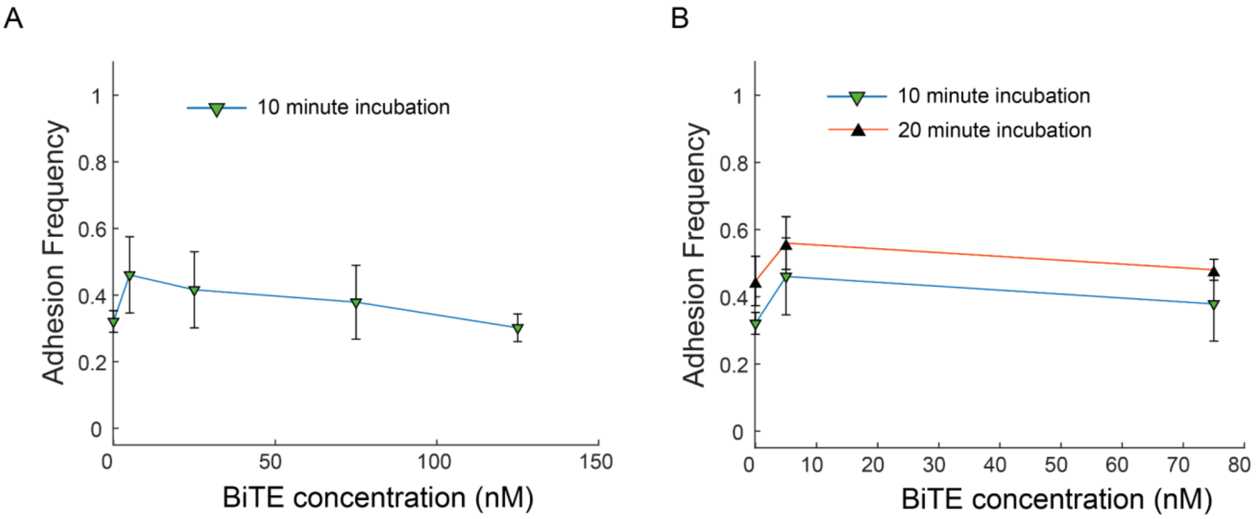

Figure S13. Fraction adhesion of Jurkat T-cell strength on BiTE-treated Nalm6 cell monolayer. A) Adhesion frequency of Jurkat cells as a function of BiTE concentration used to treat the monolayer before attachment. No significant differences were observed over the concentration range tested. Error bars represent the standard deviation over different trials. N_{trials} = [6,4,3,5,3,4], N_{cells} = [6011, 3473, 3485, 5238,3431, 5040] B) Adhesion frequency at two different attachment times and three BiTE concentrations. Error bars represent the standard deviation over different trials. 20 minute incubation numbers: N_{trials} = [4,5,3], Total cells N_{cells} = [4427,4675,2703]

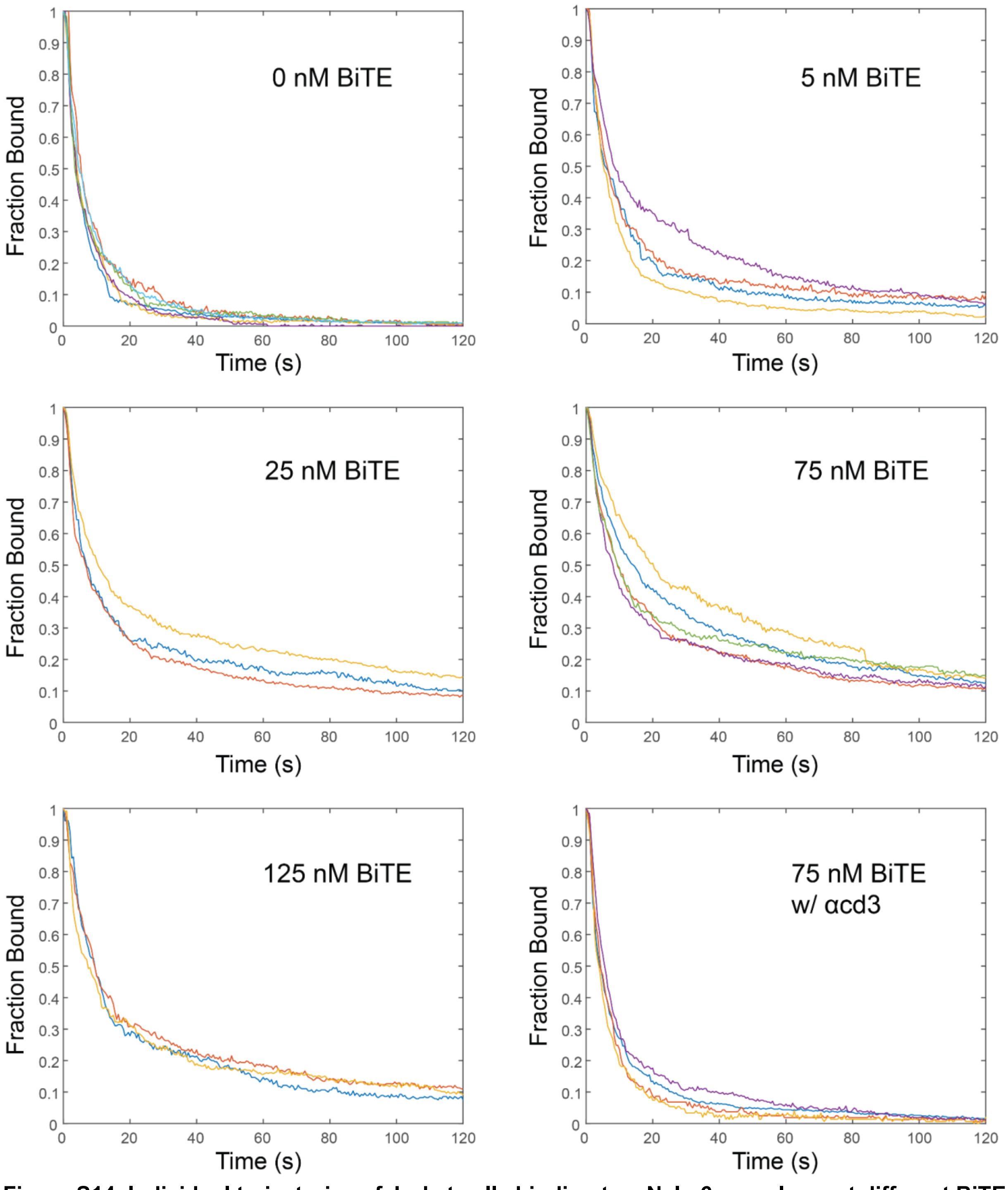

Figure S14. Individual trajectories of Jurkat cells binding to a Nalm6 monolayer at different BiTE concentrations with a 10 minute incubation. N_{trials} = [6,4,3,4,5,3,4], Total cells N_{cells} = [1946,1578, 1447, 1909, 1036, 1138]

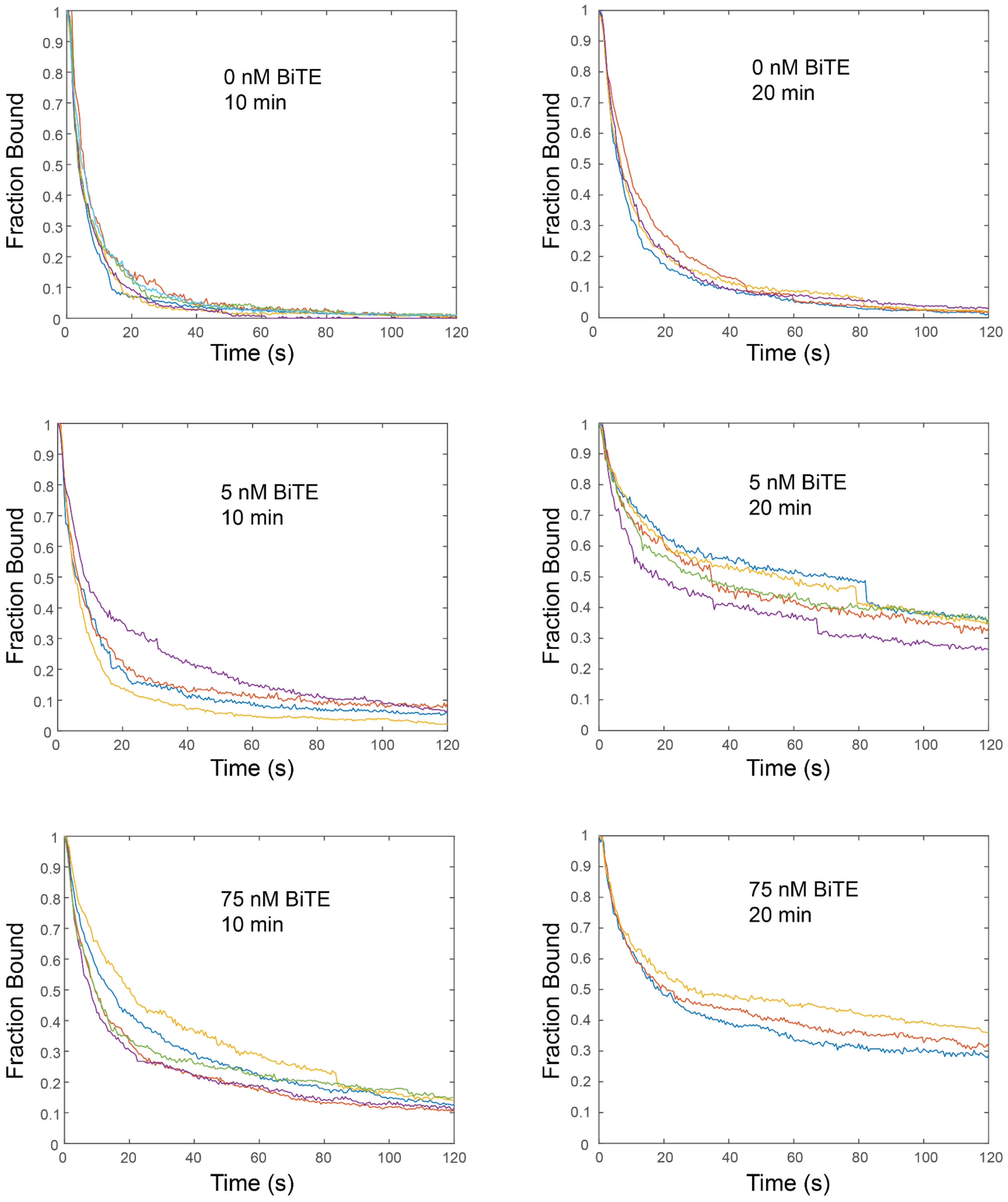

Figure S15. Individual trajectories of Jurkat cells binding to a Nalm6 monolayer with and without BiTE at different incubation times. Data listed: [0 nM 10 min, 5 nM 10 min, 75 nM 10 min, 0 nM 20 min, 5 nM 20 min,75 nM 20 min]. Number of trials: N_{trials} = [6,4,5,4,5,3], Total cells N_{cells} = [1946,1578, 1909, 1946, 2650,1319]

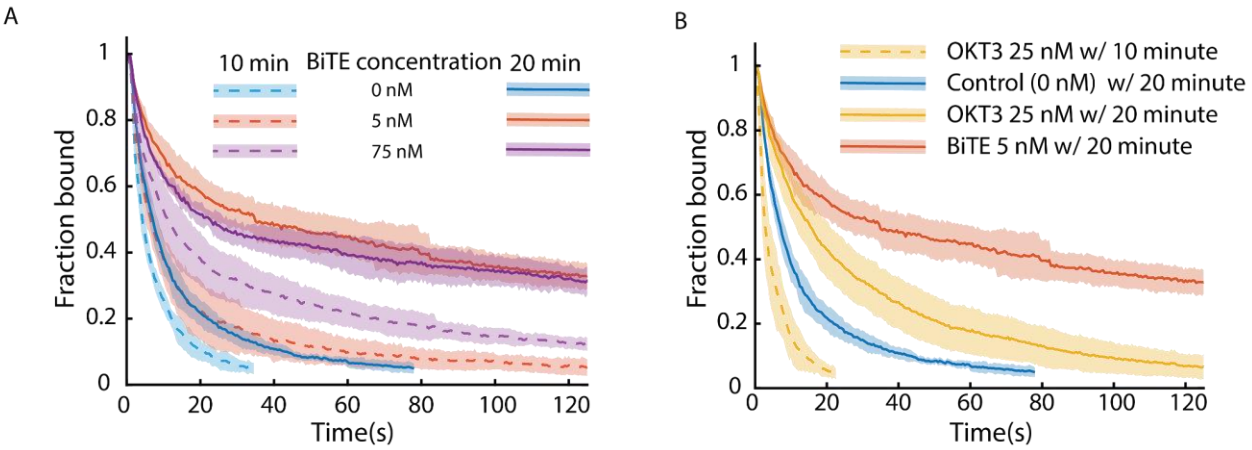

Figure S16. Cell-cell detachment curves at different BiTE concentrations and contact durations A) Time curves of Jurkat cells bound on Nalm6 monolayer at 10 and 20 minute attachment times and at three different BiTE concentrations (0 nM, 5nM, 75nM) with 4 pN/s force ramp. [0 nM 10 min, 5 nM 10 min, 75 nM 10 min, 0 nM 20 min, 5 nM 20 min,75 nM 20 min]. Number of trials: N_{trials} = [6,4,5,4,5,3] N_{cells} = [1946,1578, 1909, 1946, 2650,1319] B) Time curves of Jurkat cells bound on Nalm6 monolayer looking at the impact of incubation with anti-CD3ε antibody OKT3 using different attachment times. OKT3 was supplied at 25 nM and compared to previous BiTES and control data. Data listed as [OKT3 25 nM 10 min, Control (0 nM), OKT3 25 nM 20 min, BiTE 5 nM 20 min]. Number of trials: N_{trials} = [5,4,6,5] Total cells N_{cells} = [704,1946,1914,2650]

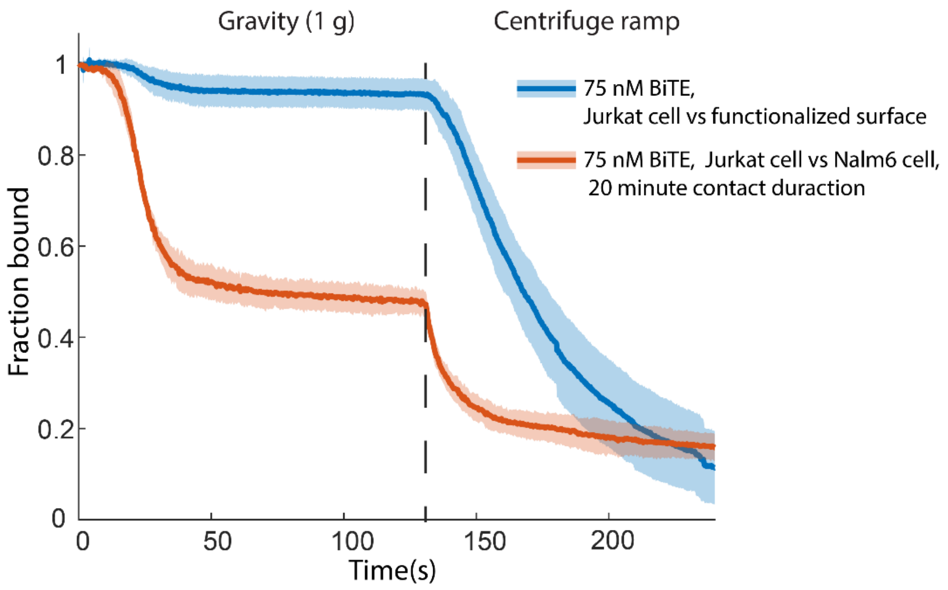

Figure S17. Comparison of detachment curves between cell–surface and cell–cell measurements. The blue curve represents Jurkat T-cell detachment from a surface functionalized with 75 nM BiTE. The curve includes an initial 2-minute interval under gravity before the centrifuge was started (dotted line), which removed non-adherent cells, followed by a 4 pN/s force ramp up to 500 pN. Few cells detached under gravity, but most unbind under applied force, with the curve trending toward 0% bound. In contrast, the red curve shows Jurkat cell detachment from a Nalm6 B-cell monolayer, also incubated with 75 nM BiTE for 20 minutes prior to measurement. Here, approximately half of the cells detached under gravity alone, but a distinct population resisted detachment under increasing force, with the curve plateauing near 20% bound. The differences between these detachment profiles are discussed in Section 4.1. The BiTE concentrations reflect different contexts—solution phase versus surface-bound—so direct comparisons of absolute binding levels are difficult. The intention is to highlight general trends in binding behavior between the two types of experiments.

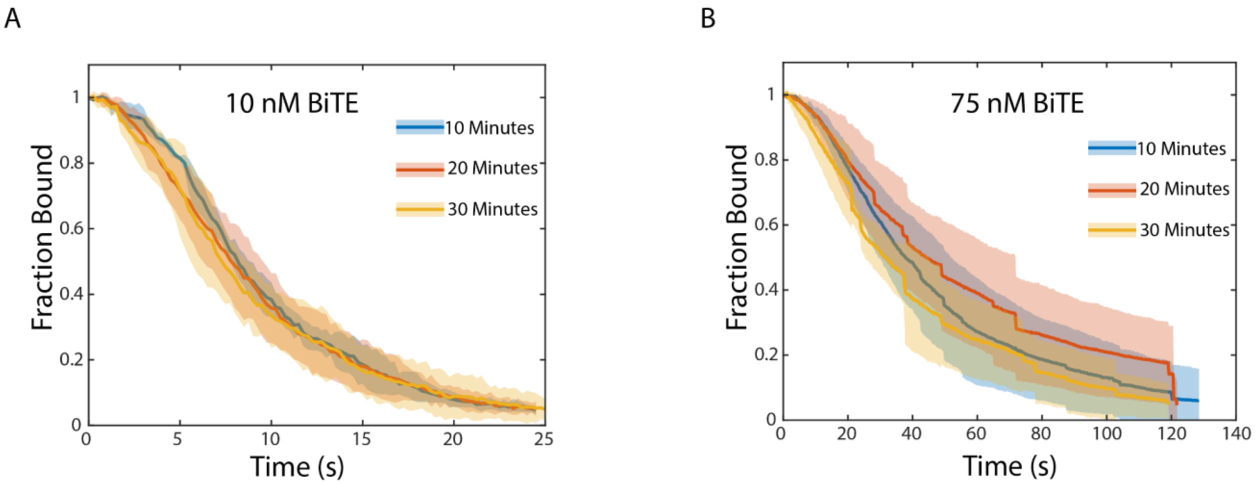

Figure S18. **Ramping curves of Jurkat T cells binding to a BiTE-functionalized surface with the specified BiTE surface concentration preparation measured using a 4 pN/s ramp.** Cells were allowed to incubate on the surface for a different time, (10, 20, or 30 minutes). No difference was observed between the different incubation times for the cell-protein measurements. 10 nM BiTE - Number of trials: N_{trials} = [3,3,3], N_{cells} = [1791,2387,2544]. 75 nM BiTE - Number of trials: N_{trials} =[4,5,3], Total cells N_{cells} = [4163,4920,3546]

Table 1: Fluorescence Centrifuge Force Microscope Updated Parts List

item #	Vendor	part #	Description	qty
1	Thorlabs	S05LEDM	SM05-Threaded Mount for LED	1
2	Thorlabs	SM1T1	SM1 (1.035"-40) Coupler	1
3	Thorlabs	SM05RR	Retaining Ring for excitation filter	1
4	Thorlabs	SM05LTRR	Retaining Ring for excitation filter with rubber o-ring	1
5	Thorlabs	SM1LTRR	Retaining Ring for emission filter with rubber o-ring	1
6	Thorlabs	LEDRGBE	627.5/525/467.5 nm Tri-Color LED	1
7	Thorlabs	SM1A6T	Diffuser and sample cell mount	1
8	SI Howard Glass Co	B-270	Ø 25 mm, 0.9 mm Thick	1
9	Kapton Tape	PPTDE-3	Sample Cell Assembly	1
10	Neuvitro	H-18-PLL	Coverslips with poly-L-lysine 18mm diameter #1 thickness	1
11	VWR	63782-01	Gold Seal, #1 19 mm coverglass	1
12	Thorlabs	SM1L03	Sample Holder, SM1 Lens Tube, 0.3" Thread Depth	1
13	Thorlabs	SM1V05	Focusing Ø1" SM1 Lens Tube	1
14	Edmund Optics	86-815	Olympus PLN 20X Objective, 0.40 NA, 1.2 mm WD	1
15	Thorlabs	SM1A3	Objective Adaptor with External SM1 Threads and Internal RMS Threads	1
16	Thorlabs	AC254-100	Tube Len, f=100.0 mm, Ø1" Achromatic Doublet, ARC: 400-700 nm	1
17	Thorlabs	SM1RR	Tube Lense SM1 Retaining Ring	2
18	Thorlabs	SM1M20	Objective SM1 Lens Tube Without External Threads, 2" Long	1
19	Thorlabs	SM1A6T	Adaptor with External SM1 Threads and Internal SM05 Threads, 0.40" Thick	2
20			Custom made turning block, aluminum	1
21	Thorlabs	PFE10-P01	Turning Mirror, 1" Silver Elliptical Mirror, 450 nm - 20 µm	2
22	Thorlabs	SM1NT	Camera SM1 (1.035"-40) Locking Ring, Ø1.25" Outer Diameter	1
23	Thorlabs	SM1A9	Camera Adaptor with External Cmount Threads and Internal SM1 Threads	1
24	Teledyne FLIR	BFS-PGE-88S6M-C	Sony IMX267 CMOS sensor, 4096 x 2160 resolution, 3.45x3.45 um pixel size	1

25	IMC Network	855-10734	MiniMc-Gigabit Twisted Pair to Fiber Media Converter	1
26	PrinceTel	MJX	Fiber Optic Rotary Joint	1
27	Chroma	89402x	Multiband pass excitation filter, unmounted, 12.5 mm diameter	1
28	Chroma	89402m	Multiband pass emission filter, unmounted, 25 mm diameter	1
29	TT Electronics	OPB732	Infrared LED and Phototransistor Long Distance Reflective Switch for RPM measurement	
30	National Instruments	NI USB-6008	DAQ board used to connect photo switch and computer for rpm measurement	1
31	Adafruit	1903	PowerBoost 500 Basic - 5V USB Boost @ 500 mA from 1.8V+	2
32	Adafruit	3500	Adafruit Trinket M0 microcontroller	1
33	Amazon		Batteries 2500 mAh TR 14500 3.7v Li-ion	6
		Other	3D printed battery pack, centrifuge bucket, wires	

Table 2:

**Spec.**	**CFM**
Applied force/cell	0 ~ 500 pN* (3,000 rpm)
Linear loading rate	Up to ~20 pN/s
Measurement cells # (in the field of view)	~1,000 cells/run
Fluorescent image	Up to 2 fluorescent colors
Image resolution	170 x 170 nm/pixel
Frame speed	8 frames/sec
Sample chamber	Disposable

*Depending on the cell size and density
