# Supplementary material for: Probing the Dynamic Strength of Biomolecular Interactions with Single-Cell Centrifugation
Source: ACS Cent Sci. 2025 Aug 26;11(10):1946–58. doi: 10.1021/acscentsci.5c00648 (PMC12550632; doi:10.1021/acscentsci.5c00648)
Supplement: Supplementary file 3 [file oc5c00648_si_003.pdf]

oc-2025-006488.R1

Name: Peer Review Information for "Probing the Dynamic Strength of Biomolecular Interactions with Single-Cell Centrifugation"

First Round of Reviewer Comments

Reviewer: 1

Comments to the Author

Probing the Dynamic Strength of Biomolecular Interactions with Single-Cell Centrifugation

This study presents exciting methodological advances in the execution and analysis of highthroughput adhesion experiments using the centrifuge force microscope (CFM). The CFM's capabilities have been expanded here by implementation of two-color fluorescence imaging, which enables the authors to inspect the presence and/or force-dependent detachment of two different objects such as two cell types during centrifugation runs. The authors demonstrate the advantages of this setup by characterizing both the adhesion frequencies as well as the detachment forces in two types of interactions, that is, interactions between (two types of) cells and surface-immobilized adhesion proteins (BiTE molecules), and BiTE-mediated interactions between these two cell types themselves.

This study should be of great interest to chemists in general and especially exciting to researchers working in the areas of single-cell and single-molecule interactions. The adhesive strength of such interactions is critical to many biomedical processes, and it is bound to continue to attract increasing attention by biochemists, biomedical engineers, and the pharmaceutical industry.

The experiments conducted in this study appear thorough, and their analysis seems sound. A highlight worth emphasizing is that the large amount of data acquired in these high-throughput experiments allows the authors to infer mechanistic insights into the studied interactions – a significant improvement over previous avidity studies that usually are limited to descriptive or correlative research. The authors' results largely support the conclusions and are well illustrated in the figures. Most of the manuscript is written clearly,

but the language suffers from a drop in quality toward the end of the manuscript that should be improved through thorough editing.

Comments and questions:

- Regarding the research presented, I only have one concern. If I understand the analysis correctly, it assumes that the cells are rigid spheres (as implied below equation 1 on page 5). However, the interior of these cells behaves more like a highly viscous fluid than a rigid solid. Not only does it seem likely that the adherent cells will spread somewhat during deposition, but I would expect that in response to the centrifugal force, their deformability will cause them to "peel" off the contact area until the force is shared between bonds along a supposedly irregular rim of the remaining contact region. Assuming the same density of adhesive bonds, the number of such load-bearing molecular attachments will depend on the actual softness of a cell. So wouldn't an alternative interpretation of differences in the attachment strengths between different cell types be the possibility that the softness of the cells is different? I realize that this would be hard to characterize experimentally, but hopefully, the authors can comment on this potential uncertainty in their interpretation.

Suggestions:

- Use of "force spectroscopy" throughout: please, reconsider. The term "force spectroscopy" has been widely used—unfortunately and incorrectly—in the context of "single-molecule force spectroscopy". In that field, it is meant to account for the fact that the characterization of

thermally susceptible molecular transitions requires mechanical tests at a broad spectrum of either clamping forces or force-loading rates. Clearly, "spectroscopy" in such a context should be replaced by "spectrometry", for the same reason that chemists have abandoned the use of "mass spectroscopy" many years ago (see Wikipedia).

- Fig. 2: I was somewhat confused by the time values included here. They created the impression of representing periods of time during which a certain RPM was maintained, as would be the case in force-clamp experiments. The authors should clarify the meaning of all time points (some appear to be intervals, others more or less random time points within the applied force ramp).
- In-line symbols including subscripts need to be formatted properly throughout the text and in figure captions.

- Between lines 40 and 41 on page 6: remove the superfluous word “Immunology”.
- Line 7 on page 3: what is meant by “cell avidity strength”?
- Caption of Fig. 1C, “Cells attach on one side with poly-L-lysine ...”: where do the cells get the PLL from? Do you mean, “Cells attach on one side to previously deposited poly-L-lysine ...”?
- Caption of Fig. 3 and elsewhere: I found the use of “trajectory” and “unbinding trajectories” for curves showing the time-dependent fraction of bound cells confusing and misleading.
- ~Line 20 on page 7, “... lifetime τ of the cells increases as more bonds are added”: who adds bonds?
- ~Line 10 on page 9, “We applied force to capture potential adhesion dynamics by measuring binding at discrete time points along a cell’s trajectory.”: Possibly due to the use of “trajectory”, I can’t make sense of this sentence.
- Page 9 and elsewhere: “Attachment time” in my understanding refers to a point in time but is used here to refer to a time period, I believe. Consider replacing it with “contact duration” or something similar.
- ~Line 50 on page 9: What exactly is meant by “BiTE binding”?
- ~Line 1 on page 10: What is meant by “BiTE dependence on adhesion frequency”?
- ~Line 8 on page 10: Reword “adhesion frequency alone struggles to distinguish binding differences”.
- Page 10, multiple places: “time dependence of adhesion” and “time dependence” needs to be defined clearer.

I don’t think the above list is complete. I encourage the authors to pay careful attention to ambivalent wording and use of in-house jargon when editing the text.

Reviewer: 2

Comments to the Author

In the paper titled “Probing the Dynamic Strength of Biomolecular Interactions with Single-Cell Centrifugation” Bergal et al. present their methodology for using centrifuge force microscopy (CFM) to probe the strength of cell-to-cell and cell-to-receptor interactions under relevant loads while monitoring fluorescence from the cells. They report on their findings with T cells and B cells engaging with a two receptor BiTE construct to demonstrate the effectiveness of their techniques.

The findings in this paper are novel and show much promise for the field of mechanobiology demonstrating the power of this new method. Of particular interest was the way that this methodology allows for a uniform force application to measure thousands of cells simultaneously with control over force as well as fluorescence. The authors do an excellent job of parameterizing the kinetics of the cell adhesion, which is complex given the changes in number of adhesions during the experiment and changes in cell activation in response to binding. They also are rigorous in showing the control over force and loading rate which are critical to these types of experiments properly. As it stands, the manuscript is thoughtful, well written, and logically sound with interest to a broad readership.

While the paper is complete and polished to a level that could be published as is, below are suggestions that would assist the reader and further strengthen the paper. It is understood that a number of these might be in the category of “appropriate for future work”.

While the preparation conditions for BiTE are detailed as concentrations, it would be good if some of the lower ones were mapped to the number of molecules in a given area. This might be straightforward to do on a different microscope by labeling BiTE with a single molecule resolution probe.   If possible, this would be useful for the readership.

Page 3: In the introduction, the readers could benefit from a little more background on the cells that are used in this experiment. While immunologists will most likely know about CD3e and CD19, it would be helpful to include a sentence or two noting what they do. For example, that CD19 is a marker for leukemia in B cells, and that it is also a part of the co-

receptor that regulates B cell activation thresholds. Additionally, note that CD3e is a member of the T cell receptor complex and binding may stimulate activation when bound to another species. This will be especially helpful to those less experienced with these immune cell systems.

Page 4: In the methods section, it wasn't clear that the Jurkats had been labeled in the earlier steps. It became apparent later in the paper during the results but consider adding a sentence to the methods explaining that both cell populations were dyed with different stains. It is also still unclear as to which cell line receives Cell Trace CFSE vs far red dye, so this could also be made more apparent in the methods.

Details on the fluorophore properties would also be helpful to include such as the peak wavelength of excitation. A reader might be selecting which sort of fluorophores might work in this setup. Relevant to this point are the spectral properties of the diode and filters. At present they will need to dig a little to determine where the diode emits and how this matches with the bands of the excitation and emission filters from Chroma.

Details of the excitation and emission filters are available form Chroma by looking up the part specifications. It would be good to note these specific wavelength bands directly in the supplemental for convenience of the reader and in case access to the transmission spectra is not available.

Page 6: There is a typo on line 40/41. The word "immunology" is a leftover at the end of the sentence.

There are some design scope and choices that would be good for the reader to benefit from the author's expertise. For the CFM setup, page 3, a 20X objective is used. The field of view is likely limited by the camera sensor size, how much additional field of view is available? This would be good to know if someone chose a different camera or wanted to view more cells. While the cells can clearly be resolved, what is the effective resolution? Likely even smaller objects can be seen, again useful for those setting up experiments on other systems. Related to this point, it would be good to note why the 20x was selected, perhaps a balance between signal available from each cell, and how many cells one would like to visualize in one image. This would help in explaining why a 20x was chosen instead of a 10x or the earlier 40x.

There may be other useful details such as the total mass of the microscope as configured here.

The details of the use of the microcontroller to toggle the LED through camera triggering signals are interesting. Is there a way to control the intensity of the LED? It wasn't immediately obvious that this whole circuitry is contained within the rotating part of the microscope. This makes sense to dedicate the fiber to just transmit camera frames. In controlling the centrifuge, it isn't clear how the ramp is controlled but presumably through the centrifuge control panel.

The flow cell preparation is clever. It is not obvious though how to exchange fluids as there is no clear "in" and "out" port. It appears that there is just a well that is filled and rinsed through the various stages. More details would be useful. For example, on page 4 line 13 "The excess cells were removed by flipping the chamber upside down in PBS for one minute.." Is this done in a beaker by dunking the chamber (and exposed tape) in a volume of PBS? What sort of volume of PBS is used. Later it appears that media is exchanged, is this done through a similar dunking method, or is the volume carefully pipetted out of the well? Perhaps some cartoons in the supplemental would help, some photos of a few of the steps or a short, annotated video. Additionally, if the tape is dunked, are there any challenges with the tape sticking/sealing after it is exposed to buffer? Does one dry off the tape somehow before final sealing?

Discussion, first paragraph "The coexistence of a large population with weak adhesion and a smaller subset with much stronger adhesion". This could be due to stochastics associated with cell landing and how they happen to have their receptors organized during this process. There might be dramatic differences in the number of receptors depending on which part of the cell binds the surface.

The timepoints associated with when the centrifuge begins and ends the ramp are very well defined. Another relevant timepoint however is when cells bind the surface or the other cell possibly initiating activation. Is it possible to define this time as well? If for instance T cell activation begins at this point due to binding or forces exerted from within the cell, one might want to know how well controlled or known this time point can be defined. It would

benefit the reader if the authors commented on how well one might control and define the initial binding relative to reasonable times associated with assembling and mounting the slide within the microscope.

Author's Response to Peer Review Comments:

Dear Editors and Reviewers at ACS Central Science,

We thank you for your positive and constructive evaluation of our manuscript. We greatly appreciate your assessment of our work as being in the Top 1% regarding “quality of experimental data, technical rigor”, “broad interest to other researchers” and “novelty”, as well as your recommendation to “Publish in ACS Central Science after minor revisions noted.” (Reviewers #1 and #2). We particularly appreciate your comments highlighting that “this study presents exciting methodological advances in the execution and analysis of high-throughput adhesion experiments using the centrifuge force microscope (CFM)” (Reviewer #1), and that “the findings in this paper are novel and show much promise for the field of mechanobiology demonstrating the power of this new method” (Reviewer #2). We also appreciate the detailed comments and suggestions, which we have addressed through new figures, text, and experiments, as described point-by-point below. Thank you once again for your thoughtful and detailed feedback, which has helped us further improve the manuscript.

Sincerely,

Wesley P. Wong, Ph.D.
Associate Professor
Harvard Medical School

--

Reviewer 1:

Probing the Dynamic Strength of Biomolecular Interactions with Single-Cell Centrifugation. This study presents exciting methodological advances in the execution and analysis of highthroughput adhesion experiments using the centrifuge force microscope (CFM). The CFM’s capabilities have been expanded here by implementation of two-color fluorescence imaging, which enables the authors to inspect the presence and/or force-dependent detachment of two different objects such as two cell types during centrifugation runs. The authors demonstrate the

advantages of this setup by characterizing both the adhesion frequencies as well as the detachment forces in two types of interactions, that is, interactions between (two types of) cells and surface-immobilized adhesion proteins (BiTE molecules), and BiTE-mediated interactions between these two cell types themselves.

This study should be of great interest to chemists in general and especially exciting to researchers working in the areas of single-cell and single-molecule interactions. The adhesive strength of such interactions is critical to many biomedical processes, and it is bound to continue to attract increasing attention by biochemists, biomedical engineers, and the pharmaceutical industry.
The experiments conducted in this study appear thorough, and their analysis seems sound. A highlight worth emphasizing is that the large amount of data acquired in these high-throughput experiments allows the authors to infer mechanistic insights into the studied interactions – a significant improvement over previous avidity studies that usually are limited to descriptive or correlative research. The authors' results largely support the conclusions and are well illustrated in the figures. Most of the manuscript is written clearly, but the language suffers from a drop in quality toward the end of the manuscript that should be improved through thorough editing.

Thank you again for the positive assessment of our manuscript, and for your constructive suggestions. We have edited the manuscript as requested to further improve the clarity of the writing.

Comments and questions:

- Regarding the research presented, I only have one concern. If I understand the analysis correctly, it assumes that the cells are rigid spheres (as implied below equation 1 on page 5). However, the interior of these cells behaves more like a highly viscous fluid than a rigid solid. Not only does it seem likely that the adherent cells will spread somewhat during deposition, but I would expect that in response to the centrifugal force, their deformability will cause them to "peel" off the contact area until the force is shared between bonds along a supposedly irregular rim of the remaining contact region. Assuming the same density of adhesive bonds, the number of such load-bearing molecular attachments will depend on the actual softness of a cell. So wouldn't an alternative interpretation of differences in the attachment strengths between different cell types be the possibility that the softness of the cells is different? I realize that this would be hard to characterize experimentally, but hopefully, the authors can comment on this potential uncertainty in their interpretation.

Thank you for raising this important point and for your thoughtful discussion. We used the assumption of spherical cells to estimate cell volume and approximate the force applied at different RPMs, rather than to explicitly model cell deformation during detachment. However, we fully agree that cell softness (mechanical compliance) can significantly influence how the membrane responds to force, affecting the membrane contact area and the transmission of force to membrane-bound receptors. These mechanical considerations could indeed influence the assumptions underlying our kinetic modeling and the receptor binding comparison discussed in Section 3.3. For instance, a more compliant cell may experience uneven stress distributions, which could alter the unbinding kinetics. Even among cells with similar compliance, variations in membrane spreading could change the effective contact area and the measured avidity.

We have revised our discussion in Section 3.3 accordingly, and have added a note on the potential importance of cell stiffness and other material properties:

"It is important to note that our CFM cell adhesion measurements represent an aggregate signal influenced by multiple factors, including receptor affinity, receptor density, membrane contact area, receptor spatial distribution, and cell mechanical compliance. Thus, the observed differences in cell avidity may also reflect additional contributions from properties such as cell stiffness. Future studies could systematically investigate these additional parameters, potentially incorporating detailed analyses of cell shape and deformation at the single-cell level."

Suggestions:

- *Use of "force spectroscopy" throughout: please, reconsider. The term "force spectroscopy" has been widely used—unfortunately and incorrectly—in the context of "single-molecule force spectroscopy". In that field, it is meant to account for the fact that the characterization of 1thermally susceptible molecular transitions requires mechanical tests at a broad spectrum of either clamping forces or force-loading rates. Clearly, "spectroscopy" in such a context should be replaced by "spectrometry", for the same reason that chemists have abandoned the use of "mass spectroscopy" many years ago (see Wikipedia).*

Thank you for this suggestion. We agree that the historical acceptance of the term "force spectroscopy" is somewhat unfortunate, although it remains the standard terminology in the field. To acknowledge this point we have added the term "force spectrometry" to the introduction as a more technically precise alternative. However, to maintain clarity and accessibility for readers from the single-molecule biophysics community, we have retained the conventional term elsewhere in the manuscript.

- *Fig. 2: I was somewhat confused by the time values included here. They created the impression of representing periods of time during which a certain RPM was maintained, as would be the case in force-clamp experiments. The authors should clarify the meaning of all time points (some appear to be intervals, others more or less random time points within the applied force ramp).*

We appreciate the suggestion about defining the time points.

We present four representative time points: the start of the experiment when the chamber is flipped to start the gravity interval; the end of the gravity interval/start of centrifugation; a midpoint during force application when more cells have detached; and the final time point at the highest applied force, when most cells have left the surface. The images shown represent a partial field of view to illustrate how the experiment appears at each stage. In the following figure, we also provide a plot showing both the number of remaining cells and the applied force over time. Additionally, we have included a video file for readers interested in viewing the full dynamic process. We have attempted to clarify the time points shown in the text.

"We show four representative partial fields of view at key time points: (1) the start, when the chamber is flipped and gravity pulls cells away (t = 0 s); (2) just before centrifugal force is applied, when adhesion frequency is measured (t = 120 s); (3) an intermediate point under centrifugal force (t = 145 s); and (4) the final point under maximum rotation speed (t = 250 s)."

- *In-line symbols including subscripts need to be formatted properly throughout the text and in figure captions.*

Thank you for pointing this out. We have updated the figure legends and main text to ensure consistency in formatting.

- *Between lines 40 and 41 on page 6: remove the superfluous word "Immunology".*

Thank you for the comment. It has been fixed in the text.

- *Line 7 on page 3: what is meant by "cell avidity strength"?*

We have removed this phrase and clarified the sentence in question. The section now reads:

"Measuring cell-cell avidity with the CFM allows us to study complex interactions under physiological receptor densities without the need for labor-intensive protein purification.

By applying force we can quantify the strength of cell-cell adhesion, which reflects the cumulative strength of multiple receptor-ligand interactions—avidity rather than single-bond affinity. This enables us to track how adhesion evolves from initial weak binding events, such as TCR-MHC engagement, to the formation of a stable immune synapse. ”

- *Caption of Fig. 1C, “Cells attach on one side with poly-L-lysine …”: where do the cells get the PLL from? Do you mean, “Cells attach on one side to previously deposited poly-L-lysine …”?*

The figure caption has been updated to clarify the attachment scheme. The caption now reads:

“Cells are deposited onto a poly-L-lysine (PLL) functionalized coverslip before the sample chamber is sealed and loaded into the fluorescence CFM.”

- *Caption of Fig. 3 and elsewhere: I found the use of “trajectory” and “unbinding trajectories” for curves showing the time-dependent fraction of bound cells confusing and misleading.*

Thank you for pointing out the confusing terminology. To address this, we have revised the wording to “cell detachment curve.” This term is commonly used to describe the time-dependent fraction of cells or particles remaining bound under shear flow–induced detachment kinetics (Decave et al., 2002; Schwarze et al., 2019).

Decave, E., Garrivier, D., Brechet, Y., Fourcade, B., & Bruckert, F. (2002). Shear flow-induced detachment kinetics of Dictyostelium discoideum cells from solid substrate. *Biophys J*, *82*(5), 2383-2395.

Schwarze, J., Grunze, M., Karahka, M., & Kreuzer, H. J. (2019). Attachment and Detachment of Particles from a Surface under Shear Flow. *J. Phys. Chem. C*, *123*, 8153-8159.

- *~Line 20 on page 7, “… lifetime τ of the cells increases as more bonds are added”: who adds bonds?*

The sentence has been rewritten to clarify our meaning.

“The fitted population lifetime (**τ**), measured during the force-ramp protocol, generally increases across trials with higher surface receptor densities, reflecting the greater number of bonds that can form under these conditions.”

- *~Line 10 on page 9, “We applied force to capture potential adhesion dynamics by measuring*

binding at discrete time points along a cell's trajectory.": Possibly due to the use of "trajectory",I can't make sense of this sentence.

The sentence has been rewritten to improve clarity.

"Using the CFM's parallel binding measurements, we investigated whether BiTE addition affects the time-dependent adhesion between Jurkat and Nalm6 cells. To do this, we varied the contact duration (i.e., the length of time that T cells are allowed to settle on the B cell monolayer prior to chamber flipping and the start of the measurement) and measured changes in binding strength."

- *Page 9 and elsewhere: "Attachment time" in my understanding refers to a point in time but is used here to refer to a time period, I believe. Consider replacing it with "contact duration" or something similar.*

We appreciate the suggestion and have updated the term to "contact duration" throughout the text for clarity.

- *~Line 50 on page 9: What exactly is meant by "BiTE binding"?*

Thank you for the helpful comment. Upon reflection, we agree that the original sentence was unclear. We have revised it for clarity and incorporated the suggested changes.

"Comparing cell adhesion mediated by BiTE molecules in the cell-cell versus the cell-surface assays reveal significant differences in their response to force as illustrated by their characteristic detachment curves (see Figure S17). Specifically, we observed a lower overall adhesion frequency in the cell-cell experiments, but a higher proportion of cells maintaining adhesion at higher forces (Figure 3C-E, 5B, Supplemental Figure S13)."

We also added the following graphic in the supplemental to help clarify our meaning in this paragraph.

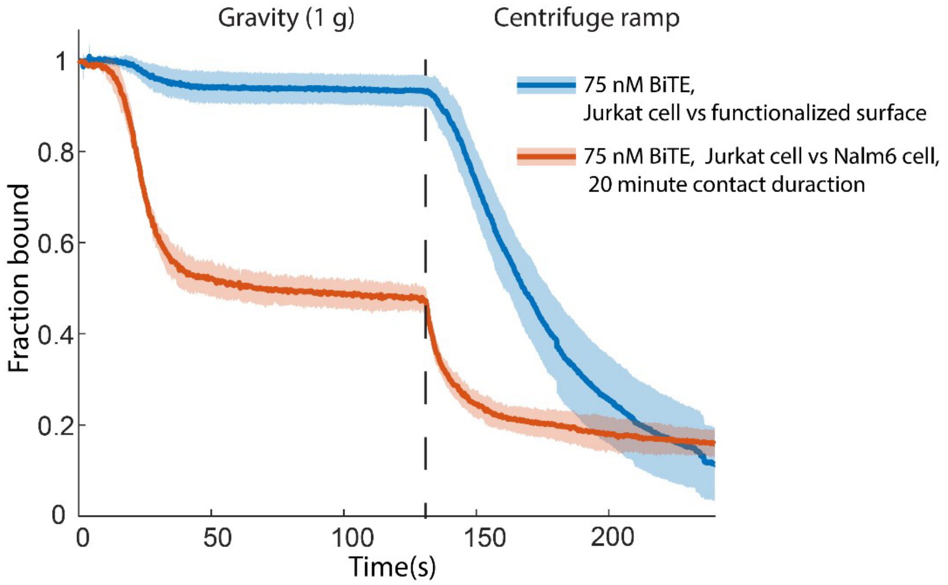

Figure S17. Comparison of detachment curves between cell–surface and cell–cell measurements. The blue curve represents Jurkat T-cell detachment from a surface functionalized with 75 nM BiTE. The curve includes an initial 2-minute interval under gravity before the centrifuge is started (dotted line), which removes non-adherent cells, followed by a 4 pN/s force ramp up to 500 pN. Few cells detach under gravity, but most unbind under applied force, with the curve trending toward 0% bound. In contrast, the red curve shows Jurkat cell detachment from a Nalm6 B-cell monolayer, also incubated with 75 nM BiTE for 20 minutes prior to measurement. Here, approximately half of the cells detach under gravity alone, but a distinct population resists detachment under increasing force, with the curve plateauing near 20% bound. The differences between these detachment profiles are discussed in Section 4.1. The BiTE concentrations reflect different contexts—solution phase versus surface-bound—so direct comparisons of absolute binding levels are difficult. The intention is to highlight general trends in binding behavior between the two types of experiments.

- *~Line 1 on page 10: What is meant by "BiTE dependence on adhesion frequency"?*

We have reworded the sentence for clarity.

"Measuring cell-cell interactions between Jurkat and Nalm6 cells reveals minimal dependence of adhesion frequency (i.e., the fraction of cells remaining after the gravity interval) on BiTE concentration (**Supplemental Figure S13**)."

- *~Line 8 on page 10: Reword "adhesion frequency alone struggles to distinguish binding differences".*

The sentence has been rewritten for clarity.

"Given the relatively high background signal for the cell-cell binding assay, adhesion frequency alone cannot differentiate between different BiTE concentrations or contact durations. However, differences between conditions become apparent when force is applied."

- *Page 10, multiple places: "time dependence of adhesion" and "time dependence" needs to be defined clearer.*

The sentences were modified to enhance readability.

"The strong dependence of cell binding on contact duration (i.e., the length of time between T cell addition and measurement) raises the question of what mechanism drives the increase."

"Additionally, no dependence on contact duration was observed in the cell-surface assay."

I don't think the above list is complete. I encourage the authors to pay careful attention to ambivalent wording and use of in-house jargon when editing the text.

We appreciate all the feedback to improve the readability and have revised sentences to improve clarity.

Reviewer 2:

In the paper titled "Probing the Dynamic Strength of Biomolecular Interactions with Single-Cell Centrifugation" Bergal et al. present their methodology for using centrifuge force microscopy (CFM) to probe the strength of cell-to-cell and cell-to-receptor interactions under relevant loads while monitoring fluorescence from the cells. They report on their findings with T cells and B cells engaging with a two receptor BiTE construct to demonstrate the effectiveness of their techniques.

The findings in this paper are novel and show much promise for the field of mechanobiology demonstrating the power of this new method. Of particular interest was the way that this methodology allows for a uniform force application to measure thousands of cells simultaneously with control over force as well as fluorescence. The authors do an excellent job

of parameterizing the kinetics of the cell adhesion, which is complex given the changes in number of adhesions during the experiment and changes in cell activation in response to binding. They also are rigorous in showing the control over force and loading rate which are critical to these types of experiments properly. As it stands, the manuscript is thoughtful, well written, and logically sound with interest to a broad readership.

While the paper is complete and polished to a level that could be published as is, below are suggestions that would assist the reader and further strengthen the paper. It is understood that a number of these might be in the category of "appropriate for future work".

While the preparation conditions for BiTE are detailed as concentrations, it would be good if some of the lower ones were mapped to the number of molecules in a given area. This might be straightforward to do on a different microscope by labeling BiTE with a single molecule resolution probe.   If possible, this would be useful for the readership.

We appreciate the suggestion and agree that characterizing the BiTE-functionalized surface would strengthen the paper and be useful for the reader. Accurately quantifying absolute single molecules across a large surface can be experimentally challenging due to factors such as labeling efficiency, photobleaching, and detection sensitivity. As an alternative to singlemolecule microscopy, we included the following experiment and supplemental figure to characterize the surface.

"To characterize the nitrocellulose surface, we applied a fluorescently labeled antibody (ThermoFisher A-11001) at varying concentrations in 30 µl droplets. After a 1-hour incubation, we measured the residual fluorescence in each droplet on a plate reader and compared it to a control that had not contacted the coverslip. For this analysis, the decrease in fluorescence was attributed to antibody adsorbing on the surface. Using the known stock concentration, fractional change in intensity, and droplet surface area, we calculated the average surface molecule density as a function of the applied concentration (Supplemental Figure S5). This measurement accounts for the total material putatively deposited, but does not take into account the orientation of the adsorbed molecules. Thus, it represents an upper limit in the number of available binding sites at each concentration. Due to this uncertainty, the sample preparation concentration is the parameter we used when labeling different conditions."

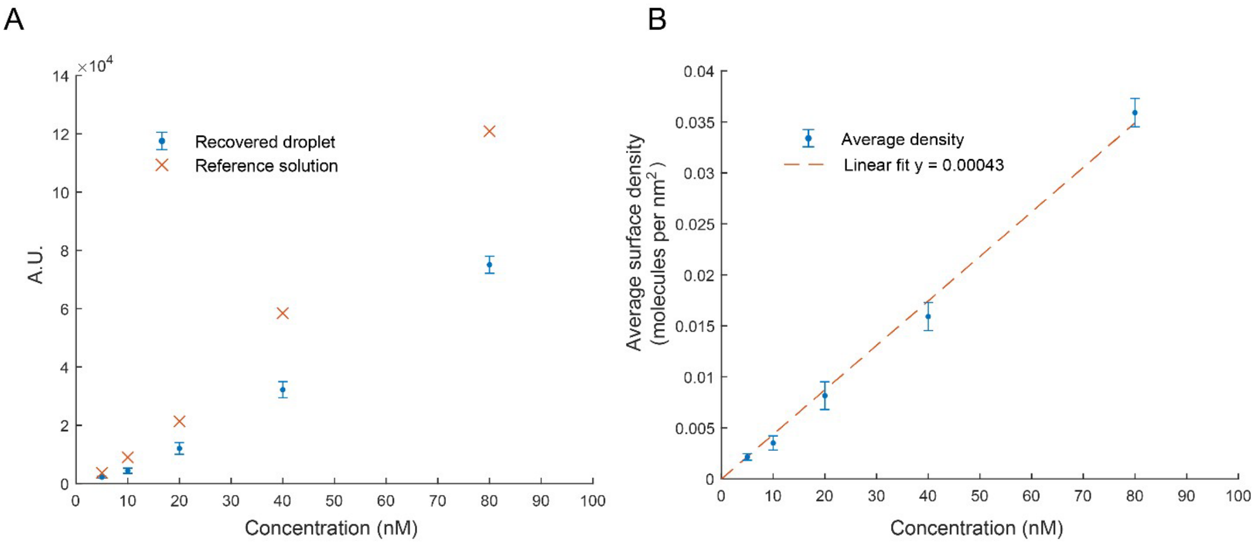

Figure S5. Surface density characterization A. Fluorescence intensity (Arbitrary Units A.U). measurements of a fluorescently labeled protein across a range of concentrations. Red '×' markers indicate reference solution measurements using a plate reader. Blue dots show measurements from recovered droplets that were first deposited onto a coverslip to allow protein adsorption, then recovered for measurement. Background fluorescence was subtracted using three PBS controls. Surface droplet measurements were repeated three times and averaged. **B.** Using data from (A), the fraction of protein retained on the surface at each concentration was calculated based on the difference between recovered droplet and reference intensity measurements. From the known droplet volume and reference concentration, the number of surface-bound molecules was calculated. Assuming a droplet contact radius of 2.5 mm, the contact area was calculated and used to determine the average surface density (molecules/nm^2) as a function of input concentration. A linear fit (y = 0.00043x, R^2 = 0.99) describes the relationship within the tested range. This estimated density serves as an upper bound on the number of available binding sites, as it does not account for molecular orientation or packing. The relationship is expected to plateau at higher concentrations due to surface saturation, but the concentrations tested remain within the linear regime.

Page 3: In the introduction, the readers could benefit from a little more background on the cells that are used in this experiment. While immunologists will most likely know about CD3e and CD19, it would be helpful to include a sentence or two noting what they do. For example, that CD19 is a marker for leukemia in B cells, and that it is also a part of the co-receptor that regulates B cell activation thresholds. Additionally, note that CD3e is a member of the T cell receptor complex and binding may stimulate activation when bound to another species. This will be especially helpful to those less experienced with these immune cell systems.

Thank you for the suggestions. As you noted, CD19 is a well-established marker for targeting Bcell malignancies in immunotherapy. It is commonly used in the development of antibody-based drugs, including blinatumomab. CD19 also plays a key role in regulating B-cell activation thresholds as part of the B-cell co-receptor complex (Wang et al., 2012). Notably, the CD19specific monoclonal antibody HD37—which serves as the origin of the CD19-binding domain in blinatumomab—has been shown to inhibit B-cell activity and proliferation (Ghetie et al., 1994). Based on this background, we included the following sentences in our manuscript.

“To demonstrate biological applications, we investigated immune cell adhesion with an FDAapproved acute lymphoblastic leukemia drug [33-35] , Blinatumomab, a bispecific T-cell engager (BiTE)[36, 37]. Blinatumomab is a single-chain variable fragment of monoclonal antibodies containing a binding site recognizing CD3ε (L2K-O7) and another site that recognizes CD19 (HD37)[28]. The CD3ε domain of the T-cell receptor (TCR) complex is known to stimulate T-cell activation signals, initiating a cascade of intracellular events that are critical for T-cell proliferation, cytokine production, and cytotoxic activity, key processes in mounting an effective immune response [38, 39]. In contrast, CD19 is a well-established marker for B-cell leukemia and a common target in immunotherapy. Notably, the CD19-specific monoclonal antibody HD37 has been shown to inhibit B-cell activity and proliferation [40]. The Blinatumomab molecules can simultaneously activate T cells via CD3ε and direct them toward $CD19^+$ B cells [41]. is. By looking at BiTE-mediated binding between Jurkat T cells and Nalm6 CD19+ B cells, we observed distinct time dynamics in the interaction profile that would be difficult to identify with other methods.”

Wang, K., Wei, G., & Liu, D. (2012). CD19: a biomarker for B cell development, lymphoma diagnosis and therapy. *Exp Hematol Oncol*, *1*(1), 36.

Ghetie, M. A., Picker, L. J., Richardson, J. A., Tucker, K., Uhr, J. W., & Vitetta, E. S. (1994). Ant-CD19 inhibits the growth of human B-cell tumor lines in vitro and of Daudi cells in SCID mice by inducing cell cycle arrest. *Blood*, *83*(5), 1329-1336.

Page 4: In the methods section, it wasn’t clear that the Jurkats had been labeled in the earlier steps. It became apparent later in the paper during the results but consider adding a sentence to the methods explaining that both cell populations were dyed with different stains. It is also still unclear as to which cell line receives Cell Trace CFSE vs far red dye, so this could also be made more apparent in the methods.

We appreciate the reviewer’s comments regarding the clarification of dye labeling for different cell types across experiments. We have added a sentence to the Methods section to specify the labeling conditions.

“The cells were spun down again, the buffer was removed, and the cells were resuspended in 10 ml RPMI+FBS. The cells were incubated for at least 20 minutes prior to imaging. For singlecell-type surface measurements, Jurkat or Nalm6 cells were labeled with CFSE dye. For cell–cell interaction experiments involving both cell types, Jurkats were labeled with Far Red dye and Nalm6 cells with CFSE.”

Details on the fluorophore properties would also be helpful to include such as the peak wavelength of excitation. A reader might be selecting which sort of fluorophores might work in this setup. Relevant to this point are the spectral properties of the diode and filters. At present they will need to dig a little to determine where the diode emits and how this matches with the bands of the excitation and emission filters from Chroma.
Details of the excitation and emission filters are available form Chroma by looking up the part specifications. It would be good to note these specific wavelength bands directly in the supplemental for convenience of the reader and in case access to the transmission spectra is not available.

We appreciate the reviewer's thoughtful suggestion. Details on the transmission wavelengths of the filter set, LED emission, and the excitation and emission spectra of the dyes have been added to Supplemental Figure 1.

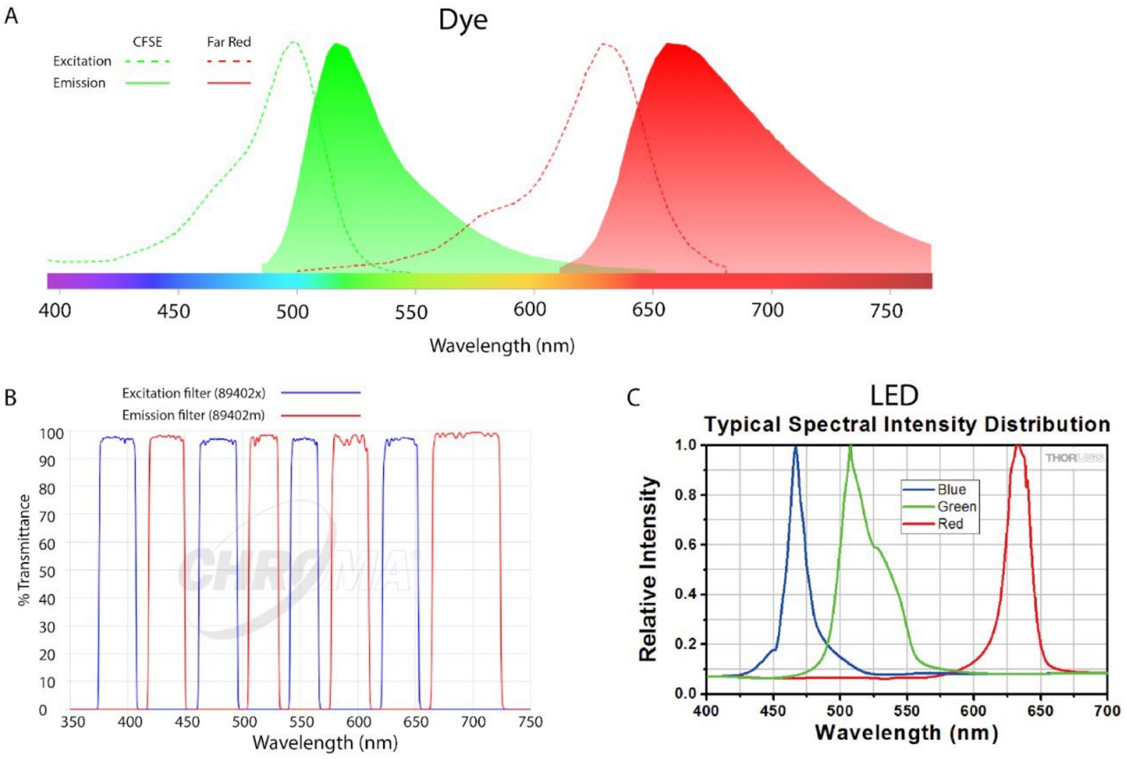

Figure S1. A. Excitation (dotted) and emission (filled) spectra for the two dyes used in the experiment (CellTrace CFSE, CellTrace Far Red). **B.** Percent transmittance spectra of excitation (89402x) and emission (89402m) filters. Data and filters from Chroma. **C.** LED emission spectra of three color RGB LED from Thorlabs. Data from Thorlabs. Full part list in Table S1.

Page 6: There is a typo on line 40/41. The word "immunology" is a leftover at the end of the sentence.

Thank you for the comment. It was fixed in the text.

There are some design scope and choices that would be good for the reader to benefit from the author's expertise. For the CFM setup, page 3, a 20X objective is used. The field of view is likely limited by the camera sensor size, how much additional field of view is available? This would be good to know if someone chose a different camera or wanted to view more cells. While the cells can clearly be resolved, what is the effective resolution? Likely even smaller objects can be seen, again useful for those setting up experiments on other systems. Related to this point, it would be good to note why the 20x was selected, perhaps a balance between signal available from each cell, and how many cells one would like to visualize in one image. This would help in explaining why a 20x was chosen instead of a 10x or the earlier 40x.

The 20x objective was chosen to balance a large field of view with sufficient image detail to clearly identify cells and facilitate troubleshooting during experiments. At this magnification, a typical cell (~10 μm in diameter) occupies approximately 50×50 pixels in the image, given a resolution of 170 nm per pixel. We have also successfully imaged beads as small as 4 μm in diameter. This additional information has been included to help other readers make design choices.

"A 20x objective was used to balance image detail and field of view (4096x2160 pixels,170 x
170 nm/pixel) enabling clear imaging of objects as small as 4 μm in diameter. Switching to a 10x objective could increase throughput by capturing roughly four times more cells per field of view. We typically targeted ~1000 cells per image to avoid clumping while maintaining high throughput, though denser packing could allow up to 5000 cells per field."

There may be other useful details such as the total mass of the microscope as configured here.

Thank you for the suggestion. We have added the total mass and geometric dimensions of the CFM to the text.

"The CFM weighs approximately 650 g and is less than 5 inches in length, making it compact enough to fit into a standard benchtop centrifuge bucket[24]"

The details of the use of the microcontroller to toggle the LED through camera triggering signals are interesting. Is there a way to control the intensity of the LED? It wasn't immediately obvious that this whole circuitry is contained within the rotating part of the microscope. This makes sense to dedicate the fiber to just transmit camera frames. In controlling the centrifuge, it isn't clear how the ramp is controlled but presumably through the centrifuge control panel.

We appreciate the questions regarding control of the CFM. The LED intensity can be modulated by adjusting the microcontroller output, which uses PWM for voltage control. However, this was not explored in depth, as the LED was typically operated at maximum brightness. We have updated the description to clarify that the circuit is contained within the centrifuge bucket and to provide additional details about RPM control.

"The LED color was controlled by a microcontroller (Trinket M0, Adafruit) housed in the 3Dprinted bucket holder, which was triggered by the CFM camera to alternate colors after each frame, enabling sequential dual-channel imaging."

"The centrifuge was modified with a control module from Thermo Fisher Scientific to enable computer control. The force ramp protocol was implemented using WinMass (Thermo Fisher Scientific) centrifuge control software, which applied small incremental steps to achieve a linear force ramp. The RPM controller script defined the loading rate based on the step size between successive commands."

The flow cell preparation is clever. It is not obvious though how to exchange fluids as there is no clear "in" and "out" port. It appears that there is just a well that is filled and rinsed through the various stages. More details would be useful. For example, on page 4 line 13 "The excess cells were removed by flipping the chamber upside down in PBS for one minute.." Is this done in a beaker by dunking the chamber (and exposed tape) in a volume of PBS? What sort of volume of PBS is used. Later it appears that media is exchanged, is this done through a similar dunking method, or is the volume carefully pipetted out of the well? Perhaps some cartoons in the supplemental would help, some photos of a few of the steps or a short, annotated video. Additionally, if the tape is dunked, are there any challenges with the tape sticking/sealing after it is exposed to buffer? Does one dry off the tape somehow before final sealing?

Thank you so much for the suggestion. We added the following description and a supplemental figure.

"After allowing cells to adsorb onto the PLL-coated coverslip for 60 minutes, excess cells were removed by inverting the sample chamber and placing it upside down into a small well containing approximately 4 mL of PBS for one minute. The chamber was then carefully lifted out—still upside down—and returned to its original orientation before adding fresh RPMI medium supplemented with FBS. The sample was incubated at 37 °C for at least 30 minutes prior to use. To preserve the sealing function of the Kapton tape, the top protective film was left in place until the lid cover glass was attached. A schematic of these procedures is now provided in Supplemental Figure 4.

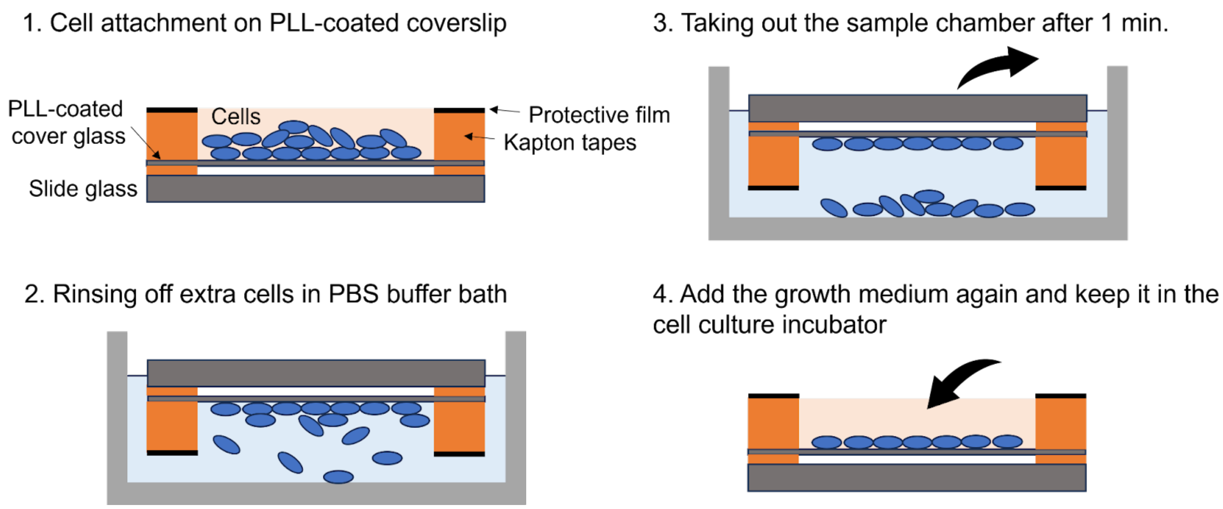

Figure S4. Schematic of Nalm6 cell monolayer formation on a PLL-coated coverslip. The process involves four steps: Step 1: A suspension of Nalm6 cells in RPMI medium (without FBS) is added to the PLL-coated coverslip. Step 2: After a 60-minute incubation to allow cell adsorption, excess cells are removed by inverting the chamber and placing it upside down in a well containing ~4 mL PBS. Step 3: The chamber is then gently lifted out, remaining inverted to avoid disturbing the attached cells. Step 4: The chamber is returned to its upright position, and fresh RPMI medium with FBS is added. To preserve the seal formed by the Kapton tape, the protective film is left intact until the top cover glass is applied."

Discussion, first paragraph "The coexistence of a large population with weak adhesion and a smaller subset with much stronger adhesion". This could be due to stochastics associated with cell landing and how they happen to have their receptors organized during this process. There might be dramatic differences in the number of receptors depending on which part of the cell binds the surface.

We appreciate this insightful comment and have added an alternative explanation in the discussion.

"The coexistence of a large population with weak adhesion and a smaller subset with much stronger adhesion suggests the presence of a distinct subpopulation with a different response to BiTE in the presence of Nalm6 B cells. Alternatively, this pattern may arise from substantial heterogeneity in surface receptor organization, potentially influenced by how cells land and establish contact."

The timepoints associated with when the centrifuge begins and ends the ramp are very well defined. Another relevant timepoint however is when cells bind the surface or the other cell possibly initiating activation. Is it possible to define this time as well? If for instance T cell activation begins at this point due to binding or forces exerted from within the cell, one might want to know how well controlled or known this time point can be defined. It would benefit the reader if the authors commented on how well one might control and define the initial binding relative to reasonable times associated with assembling and mounting the slide within the microscope.

Thank you for your valuable comments and questions. We agree that capturing the very earliest moments of T-cell activation presents a significant challenge. The onset of activation—such as calcium flux triggered by early T-cell receptor (TCR) signaling—can occur within seconds, while downstream processes like cytoskeletal rearrangement typically unfold over several minutes (Huse, 2007). In our experiments, the 10-minute contact period allows sufficient time for activation to initiate and begin progressing, though it may not fully capture the earliest molecular events.

The "contact duration" we use in the paper is the time from first contact (i.e when the T cells are added to the B cell monolayer) to when the chamber is flipped over to begin the gravity interval and the recording/experiment begins (t=0, figure 2A). In our CFM system, assembling and mounting the components, including the sample chamber, takes at least 3 minutes, which represents a lower boundary to the contact duration we can probe. To ensure consistency in activation timing across all measurements, we standardized the procedure by measuring avidity after at least 10-minute contact duration. Since T-cell activation phenomena, like immune synapse formation, are known to persist for several hours, we consider that activation signaling continues within the timeframe of our measurements (Fritzsche et al., 2017).

Looking ahead, if we are able to automate the delivery of effector cells (e.g., Jurkat cells in this study), it may become possible to observe adhesion forces during the very early stages of activation.

Huse, M., Klein, L. O., Girvin, A. T., Faraj, J. M., Li, Q. J., Kuhns, M. S., & Davis, M. M. (2007). Spatial and temporal dynamics of T cell receptor signaling with a photoactivatable agonist. *Immunity*, *27*(1), 76-88.

Fritzsche, M., Fernandes, R. A., Chang, V. T., Colin-York, H., Clausen, M. P., Felce, J. H., Galiani, S., Erlenkamper, C., Santos, A. M., Heddleston, J. M., Pedroza-Pacheco, I., Waithe, D., de la Serna, J. B., Lagerholm, B. C., Liu, T. L., Chew, T. L., Betzig, E., Davis, S. J., &

Eggeling, C. (2017). Cytoskeletal actin dynamics shape a ramifying actin network underpinning immunological synapse formation. *Sci Adv*, *3*(6), e1603032.
